# Health Care Spending After Initiating Sacubitril-Valsartan vs Renin-Angiotensin System Blockers for Heart Failure Treatment

**DOI:** 10.1001/jamahealthforum.2024.5385

**Published:** 2025-02-14

**Authors:** Catherine S. Hwang, Rishi J. Desai, Aaron S. Kesselheim, Raisa Levin, Sushama Kattinakere Sreedhara, Benjamin N. Rome

**Affiliations:** 1 Division of Pharmacoepidemiology and Pharmacoeconomics, Department of Medicine, Brigham and Women’s Hospital and Harvard Medical School, Boston, Massachusetts; 2 Division of General Internal Medicine and Geriatrics, Oregon Health and Science University, Portland; 3 Section of General Internal Medicine, VA Portland Health Care System, Portland, Oregon

## Abstract

**Importance:**

For patients with heart failure with reduced ejection fraction (HFrEF), treatment with sacubitril-valsartan, an angiotensin receptor−neprilysin inhibitor, has become increasingly preferred over angiotensin-converting enzyme inhibitors (ACE-Is) and angiotensin II receptor blockers (ARBs). However, sacubitril-valsartan is much more expensive than generic ACE-I/ARBs. It is unknown whether the high cost of sacubitril-valsartan is offset by lower spending on hospitalizations and other treatments.

**Objective:**

To compare total and out-of-pocket health care spending among Medicare beneficiaries initiating sacubitril-valsartan vs ACE-I/ARBs for HFrEF.

**Design, Setting, and Participants:**

This was a cohort study using data from Medicare fee-for-service claims with propensity score matching of Medicare beneficiaries with HFrEF. Data analysis was performed from November 2022 to December 2023.

**Exposure:**

Initiation of sacubitril-valsartan or an ACE-I/ARB. Patients were matched by propensity score based on 104 covariates, including demographic characteristics, comorbidities, baseline annual spending, and baseline use of health care services.

**Main Outcomes and Measures:**

Mean total and out-of-pocket health care expenditures during the 365 days after initiating sacubitril-valsartan or an ACE-I/ARB. Censoring for incomplete follow-up was addressed using Kaplan-Meier probability weighting. Cost differences, cost ratios, and 95% CIs were calculated using a nonparametric bootstrapping method with 500 samples drawn with replacement.

**Results:**

Among 13 755 matched pairs of Medicare patients with HFrEF (mean [SD] age, 77.5 [7.5] years; 5138 [39%] 80 years or older; 9949 females [36%] and 17 561 males [64%]), mean annual total health care spending per person was similar for sacubitril-valsartan initiators and ACE-I/ARB initiators (difference, $701; 95% CI, −$132 to $1593). Sacubitril-valsartan initiators had higher prescription drug costs (difference, $1911; 95% CI, $1704 to $2113), lower inpatient costs (difference, −$790; 95% CI, −$1468 to −$72), lower outpatient costs (difference, −$330; 95% CI, −$664 to −$11), and higher annual out-of-pocket spending (difference, $109; 95% CI, $13 to $208).

**Conclusions and Relevance:**

This cohort study found that Medicare beneficiaries initiating sacubitril-valsartan to treat HFrEF had similar total health care spending as those initiating ACE-I/ARBs; higher prescription drug spending was offset by lower inpatient and outpatient spending. However, sacubitril-valsartan use was associated with higher patient out-of-pocket costs, which may exacerbate health disparities and limit access and affordability.

## Key Points

**Question:**

How does initiation of sacubitril-valsartan affect total and out-of-pocket health care expenditures compared with initiation of other renin-angiotensin system blockers in Medicare beneficiaries with heart failure with reduced ejection fraction?

**Findings:**

In this cohort study of 13 755 propensity score−matched pairs, annual total health care spending was similar for patients initiating sacubitril-valsartan and those initiating renin-angiotensin system blockers; higher prescription drug cost of sacubitril-valsartan was offset by lower inpatient and outpatient spending. However, patient out-of-pocket costs were higher for those initiating sacubitril-valsartan.

**Meaning:**

These findings suggest that the high price of sacubitril-valsartan was not associated with differences in total health care spending, but was associated with higher patient out-of-pocket costs, a finding that has important implications for access, affordability, and equity.

## Teaser

This cohort study assesses health care costs after initiating sacubitril-valsartan compared to an angiotensin-converting enzyme inhibitor or an angiotensin II receptor-blocker in Medicare patients being treated for heart failure with reduced ejection fraction.

## Introduction

Cardiovascular disease is the leading cause of morbidity and mortality in the US.^1^ Heart failure is an important driver, affecting 6.7 million adults.^2^ In 2020, heart failure was the underlying cause or a contributing factor in the death of 85 855 and 415 922 individuals, respectively.^2^ Medicare beneficiaries represent a large proportion of the population with heart failure, with an incidence rate of 26.5 per 1000 annually.^3^

Treatment for heart failure is costly. The median (IQR) annual direct cost for heart failure care was $24 383 ($20 713 to $40 619) per patient between 2014 and 2020.^4^ Although much of this cost was related to expensive hospitalizations and emergency department visits, a substantial proportion was also due to medications. Some of these costs were passed onto patients; annual out-of-pocket costs for patients with heart failure averaged $4423 from 2014 to 2018, with medications representing the largest category of cost.^5^

The medical management of heart failure has evolved with newer and more expensive medications entering the market. Until recently, 3 classes of medications were the cornerstone of guideline-directed medical therapy for heart failure with reduced ejection fraction (HFrEF): angiotensin-converting enzyme inhibitors (ACE-Is) and angiotensin II receptor blockers (ARBs), β-blockers, and aldosterone receptor antagonists. Each has strong evidence supporting reduced mortality, morbidity, and hospitalizations.^6,7^ By 2010, members of each of these drug classes were available in low-cost generic formulations.^8,9,10^

In 2014, the Prospective Comparison of ARNI with ACE-I to Determine Impact on Global Mortality and Morbidity in Heart Failure (PARADIGM-HF)^11^ randomized clinical trial demonstrated that the combination of a neprilysin inhibitor (sacubitril) plus an ARB (valsartan) reduced heart failure hospitalizations and deaths compared to ACE-I (enalapril) monotherapy. The US Food and Drug Administration (FDA) subsequently approved sacubitril-valsartan on July 7, 2015.^12^ Since then, guidelines have transitioned toward recommending sacubitril-valsartan over traditional ACE-I/ARBs in patients with heart failure.^13^

Unlike the ACE-I and ARB medication classes, sacubitril-valsartan is the only brand-name angiotensin receptor−neprilysin inhibitor (ARNI) currently available. The annual prerebate cost of sacubitril-valsartan is $7091, compared to $192 for valsartan and $176 for enalapril.^14^ The financial burden on patients is also higher; the average out-of-pocket cost of sacubitril-valsartan among a cohort of Medicare Advantage beneficiaries was $62 per month compared to less than $3 per month for ACE-I/ARB users.^15^

Some of this higher spending may be offset by lower downstream costs if those using sacubitril-valsartan have fewer heart failure hospitalizations and other complications. However, high out-of-pocket costs for sacubitril-valsartan may also be associated with cost-related medication nonadherence that may temper the benefits observed in clinical trials. To understand how sacubitril-valsartan affects health care costs among a cohort of patients treated in routine clinical settings, we compared total and out-of-pocket health care expenditures for Medicare beneficiaries with HFrEF initiating this brand-name medication, compared to those initiating a generic ACE-I/ARB.

## Methods

The Mass General Brigham Institutional Review Board deemed the study exempt from review and waived informed consent because only deidentified claims data were used. The findings are reported in accordance with Strengthening the Reporting of Observational Studies in Epidemiology (STROBE) reporting guideline.

### Data Source and Study Design

We used insurance claims data from a 100% sample of Medicare fee-for-service beneficiaries to conduct a cohort study of individuals with HFrEF initiating sacubitril-valsartan compared to ACE-I/ARBs, from October 1, 2016 to December 31, 2019 (end of available data). Although sacubitril-valsartan was approved by the FDA in July 2015, we selected October 2016 as the start of our study to ensure that the entire study, including the 365-day baseline period, could be measured using the *International Statistical Classification of Diseases and Related Health Problems, Tenth Revision *(*ICD-10*) codes, effective since October 1, 2015.

### Cohort Definition

We identified new users of ARNIs or ACE-I/ARBs, defined as the first filled prescription of any medication in these classes after a 365-day period of continuous Medicare enrollment, during which patients had no prior use of any ARNI, ACE-I, or ARB. Medication use was identified based on generic names; we included combination products that contained an ACE-I/ARB, such as amlodipine-benazepril, amlodipine-valsartan, and losartan-hydrochlorothiazide.

We limited our cohort to those with at least 1 *ICD-10* diagnostic code for heart failure during the 365 days before sacubitril-valsartan, ACE-I, or ARB initiation. Because sacubitril-valsartan was FDA-approved for only HFrEF during the study period, we decided that the use of this medication plus a single *ICD-10* code was sufficiently specific to identify patients with HFrEF. By contrast, ACE-I/ARBs are used to treat several other cardiovascular conditions, such as hypertension. To identify patients using these medications specifically for HFrEF, we restricted this group based on a previously validated claims-based algorithm.^16^ This algorithm uses information available from Medicare claims, such as demographic characteristics and comorbidity diagnoses, to probabilistically phenotype patients into heart failure with reduced or preserved ejection fraction. We excluded individuals who filled prescriptions for sacubitril-valsartan and either an ACE-I or ARB on the same day; were younger than 65 years; were residing outside of the 50 US states or the District of Columbia; and/or had missing data for age, sex, or US Census Bureau region information. Medicare claims data used for this study excluded patients younger than 65 years; this younger population must have certain qualifying conditions or disabilities, and therefore, are less representative of the older US population with heart failure.

**Figure 1.**
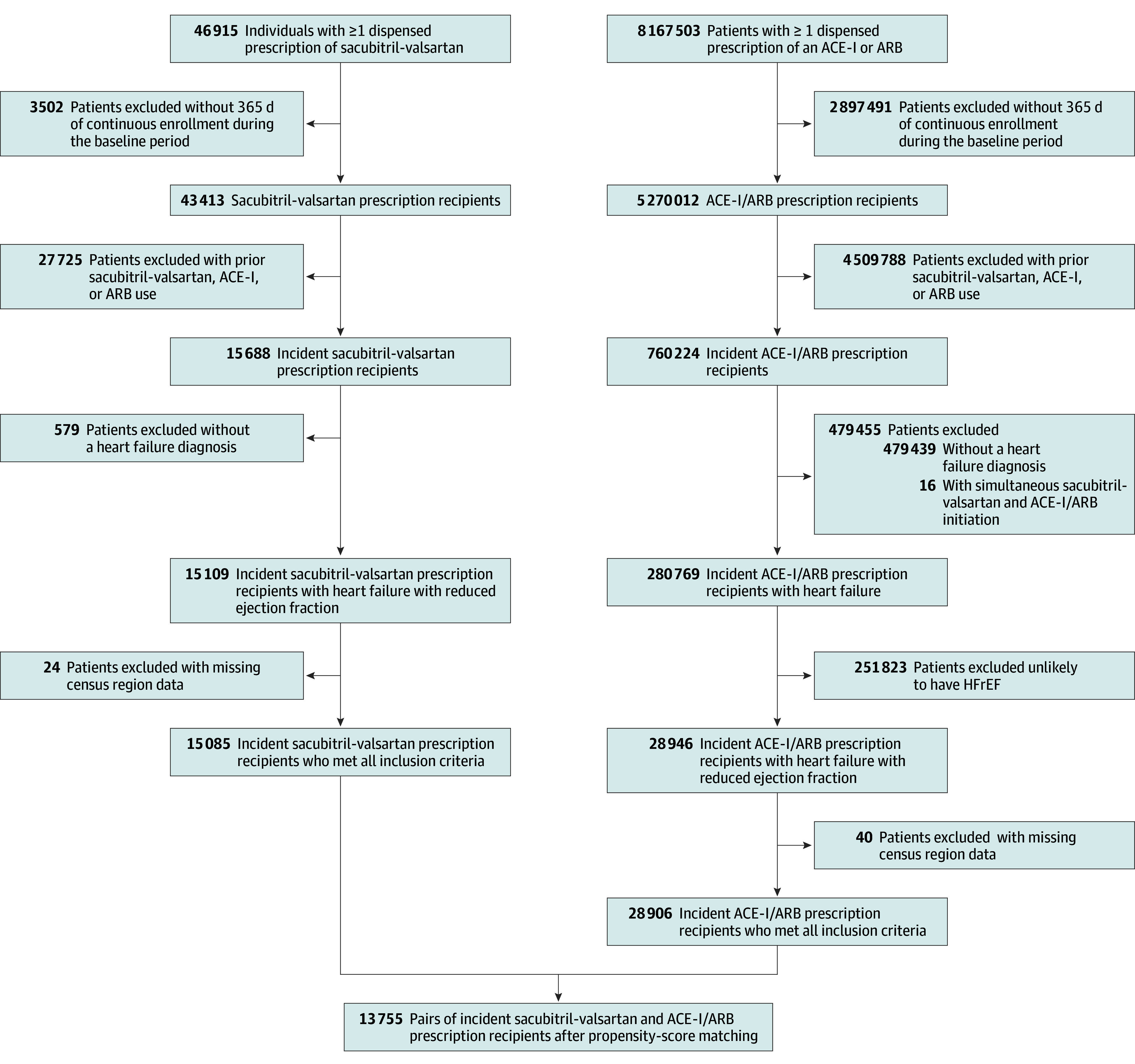
Flow Diagram of Study Participants ACE-I indicates angiotensin-converting enzyme inhibitors; ARB, angiotensin ii receptor blockers; and HFrEF, heart failure with reduced ejection fraction.

We identified 15 085 ARNI initiators and 28 906 ACE-I/ARB initiators who met all inclusion criteria (Figure 1). Before matching, the 2 groups were similar (eFigure 1 in Supplement 1), although ARNI initiators had less use of some health care services and lower baseline mean costs ($43 398 vs $56 553; eTable 2 in Supplement 1).

### Outcomes

The primary outcomes of interest were total and out-of-pocket spending during the 365 days after sacubitril-valsartan or ACE-I/ARB initiation. Total spending was defined as the sum of payments by patients, Medicare, and third parties (eg, secondary insurers). Out-of-pocket spending paid by patients included copayments, coinsurances, and deductibles. We stratified total spending by inpatient services (Medicare Part A, including hospital and skilled nursing facility admissions), outpatient services (Medicare Part B, including office visits, emergency department care, and durable medical equipment), and retail prescription drugs (Medicare Part D), as detailed in eTable 1 in Supplement 1. Furthermore, we stratified prescription drug expenditures by spending on sacubitril-valsartan, ACE-Is, and ARBs vs spending on all other prescription medications.

Reported Medicare spending on brand-name prescription drugs, such as sacubitril-valsartan, does not reflect manufacturer rebates paid after the point of sale.^17^ To address this, we performed secondary analyses in which we reduced sacubitril-valsartan spending by quarterly rebate estimates from SSR Health.^17,18,19^ We assumed that ACE-I/ARBs, all of which have generic versions available, did not have any rebates. Spending data were converted to December 2019 US dollars using the Consumer Price Index for All Urban Consumers.^20^

### Covariates

We adjusted for differences in 104 covariates (eTables 2 and 3 in Supplement 1) measured during the 365-day baseline period before sacubitril-valsartan or ACE-I/ARB initiation. These consisted of demographic characteristics, including age, sex, US census region, and qualification for Medicare low-income subsidies; comorbidities commonly associated with heart failure, including chronic kidney disease, coronary artery disease, and diabetes; baseline use of health care services, including medications, hospitalizations, emergency department visits, office visits, laboratory tests, and age-appropriate cancer screenings and immunizations; the quarter of medication initiation; and baseline annual health care spending.

### Statistical Analysis

To adjust for confounding based on differences between sacubitril-valsartan and ACE-I/ARB initiators, we identified pairs of patients matched using a propensity score.^21^ Briefly, a logistic regression model was constructed to predict the probability of sacubitril-valsartan initiation, conditional on the covariates listed in eTables 2 and 3 in Supplement 1. Based on this model, we matched sacubitril-valsartan and ACE-I/ARB initiators, using a 1:1 nearest-neighbor matching algorithm and a 0.01 caliper. We assessed the balance between the 2 groups based on standardized differences of less than 0.1 for all covariates.

We reported annual spending for the cost outcomes in the propensity score−matched cohort, stratified by exposure group. We quantified cost differences and ratios between the sacubitril-valsartan and ACE-I/ARB groups. To account for the right-skewed nature of the cost data, we used a γ distribution when constructing models to compare mean cost differences between the sacubitril-valsartan and ACE-I/ARB groups. We calculated 95% CIs using a nonparametric bootstrapping method with 500 samples drawn with replacement.

In the primary analysis, we used an a priori intention-to-treat approach, where we followed patients for as many as 365 days, regardless of whether their sacubitril-valsartan or ACE-I/ARB use was subsequently discontinued. To account for incomplete follow-up because of censoring before 365 days due to death, disenrollment from Medicare, or the end of available data, we used Kaplan-Meier probability weighting to estimate the average annual costs in the 2 exposure groups.^22^ We chose an intention-to-treat approach, rather than an as-treated approach, for the primary analyses because it more accurately reflects the realities of medication use in routine clinical practice, including treatment nonadherence-related costs.

In sensitivity analyses, we repeated the primary analyses using an as-treated approach, in which we also censored individuals who discontinued their index sacubitril-valsartan or ACE-I/ARB use or switched to a medication in the opposite group. Discontinuation was defined as lack of a dispensed prescription for sacubitril-valsartan or an ACE-I/ARB after a 30-day gap following the last filled prescription. In addition, to ensure that 500 bootstrapped samples were sufficient for precise CI estimates, we repeated the CI calculations for the main analysis using 1000 bootstraps. Finally, to identify whether the price of sacubitril-valsartan may be associated with differential effects on spending in certain high-risk populations, we performed subgroup analyses, stratifying patients by age (<80 or ≥80 years), sex (male/female), race and ethnicity (White or other race and ethnicity [Asian, Black, Hispanic, and other]), frailty (not frail/prefrail/frail), receipt of Medicare low-income subsidies (yes/no), baseline health care costs (by quartile), and whether patients had at least 1 hospitalization with heart failure as their primary diagnosis during the 365-day baseline period (yes/no). All demographic information, including race and ethnicity, was obtained from Medicare fee-for-service claims. Frailty was measured using a validated claims-based frailty index (range 0-1) previously described in the literature.^23^ We used clinically meaningful thresholds to categorize individuals into 3 frailty groups by frailty index score: not frail (<0.15), prefrail (0.15-0.24), and frail (≥0.25).^24,25,26,27^

Statistical tests were 2-tailed, and *P* < .05 was considered statistically significant. All analyses were conducted using SAS statistical software, version 9.4 (SAS Institute) from November 2022 to December 2023.

## Results

After propensity score matching, we included 13 755 pairs of patients (mean [SD] age, 77.5 [7.5] years; 9949 females [36%] and 17 561 males [64%]; 446 Asian [2%], 2747 Black [10%], 464 Hispanic [2%], 23 084 White [84%], and 769 individuals of other race or ethnicity [3%]) who were balanced across the 104 covariates in the analytic cohort. An abbreviated list of covariates is available in Table 1, and the complete list in eTable 3 in Supplement 1, with density plots in eFigure 2 in Supplement 1. Those 80 years and older composed 39% of the analytic cohort.

**Table 1.** Baseline Characteristics of Patients With Heart Failure With Reduced Ejection Fraction in Propensity Matched Cohort

Variable	Sacubitril-valsartan initiators, No. (%)	ACE-I/ARBs initiators, No. (%)	Standardized difference
Total patients, No.	13 755	13 755	NA
**Demographic characteristics**			
Female	4985 (36.2)	4964 (36.1)	−0.0032
Male	8770 (63.8)	8791 (63.9)	
Age, y			
65-69	2264 (16.5)	2343 (17.0)	−0.0154
70-74	3058 (22.2)	2996 (21.8)	0.0109
75-79	3115 (22.6)	3018 (21.9)	0.0169
80-84	2633 (19.1)	2665 (19.4)	−0.0059
85-89	1762 (12.8)	1802 (13.1)	−0.0087
≥90	923 (6.7)	931 (6.8)	−0.0023
US Census Bureau region			
Midwest	2798 (20.3)	2789 (20.3)	0.0016
Northeast	2537 (18.4)	2521 (18.3)	0.0030
South	6355 (46.2)	6373 (46.3)	−0.0026
West	2065 (15.0)	2072 (15.1)	−0.0014
Low-income subsidy recipient	3784 (27.5)	3704 (26.9)	0.0131
Comorbidities			
Atrial fibrillation	8650 (62.9)	8684 (63.1)	−0.0051
Chronic kidney disease	6329 (46.0)	6369 (46.3)	−0.0058
Coronary artery disease	3869 (28.1)	3869 (28.1)	0.0000
Diabetes	8188 (59.5)	8176 (59.4)	0.0018
Hypertension	13 144 (95.6)	13 145 (95.6)	−0.0003
Medication use			
β-Blockers	12 241 (89.0)	12 241 (89.0)	0.0000
Calcium channel blockers	3143 (22.8)	3122 (22.7)	0.0036
Digoxin	2132 (15.5)	2118 (15.4)	0.0028
Hydralazine	1297 (9.4)	1348 (9.8)	−0.0126
Loop diuretics	10 781 (78.4)	10 751 (78.2)	0.0053
Nitrates	2346 (17.1)	2384 (17.3)	−0.0073
Statins	9504 (69.1)	9492 (69.0)	0.0019
SGLT2 inhibitors	235 (1.7)	220 (1.6)	0.0085
Thiazide diuretics	1768 (12.9)	1788 (13.0)	−0.0043
**Health care utilization in past 365 d, mean (SD)**			
ED visits	2.2 (2.7)	2.3 (2.6)	−0.0237
Hospitalizations			
Heart failure as primary diagnosis	0.5 (0.9)	0.5 (0.8)	−0.0116
Heart failure not as primary diagnosis	1.0 (1.4)	1.0 (1.3)	−0.0177
Office visits			
Cardiology	10.6 (10.7)	10.8 (11.7)	−0.0165
Internal/family medicine	19.0 (27.8)	19.2 (26.9)	−0.0088
Dispensed medications			
Brand	4.2 (3.1)	4.2 (3.2)	−0.0023
Generic	10.1 (4.8)	10.2 (5.1)	−0.0198
Laboratory and imaging tests			
Echocardiography	12 191 (88.6)	12 210 (88.8)	−0.0044
Electrocardiography	12 681 (92.2)	12 683 (92.2)	−0.0005
Total cost in past 365 d, $	44 808 (48 490)	46 103 (52 989)	−0.0016^a^

Abbreviations: ACE-Is, angiotensin-converting enzyme inhibitors; ARBs, angiotensin II receptor blockers; ED, emergency department; NA, not applicable; SGLT2, sodium-glucose cotransporter-2.

^a^ Log-transformed to better represent the right-skewed distribution of cost data.

In addition to HFrEF, 28% of these Medicare beneficiaries had comorbid coronary artery disease, 46% had chronic kidney disease, and 96% had hypertension. During the 365 days before initiating the index drug, patients had a mean (SD) of 2.3 (2.6) emergency department visits, 0.5 (0.8) hospitalizations with heart failure in the primary diagnosis position, and 11.0 (11.0) office visits with a cardiologist. Mean (SD) total annual health care costs during the baseline period were $44 808 ($48 490) for sacubitril-valsartan initiators and $46 103 ($52 989) for ACE-I/ARB initiators. Overall, 45% of patients had the full 365 days of follow-up, 39% were censored due to disenrollment, and 16% were censored due to death (eTable 4 in Supplement 1).

### Total Spending

**Figure 2.**
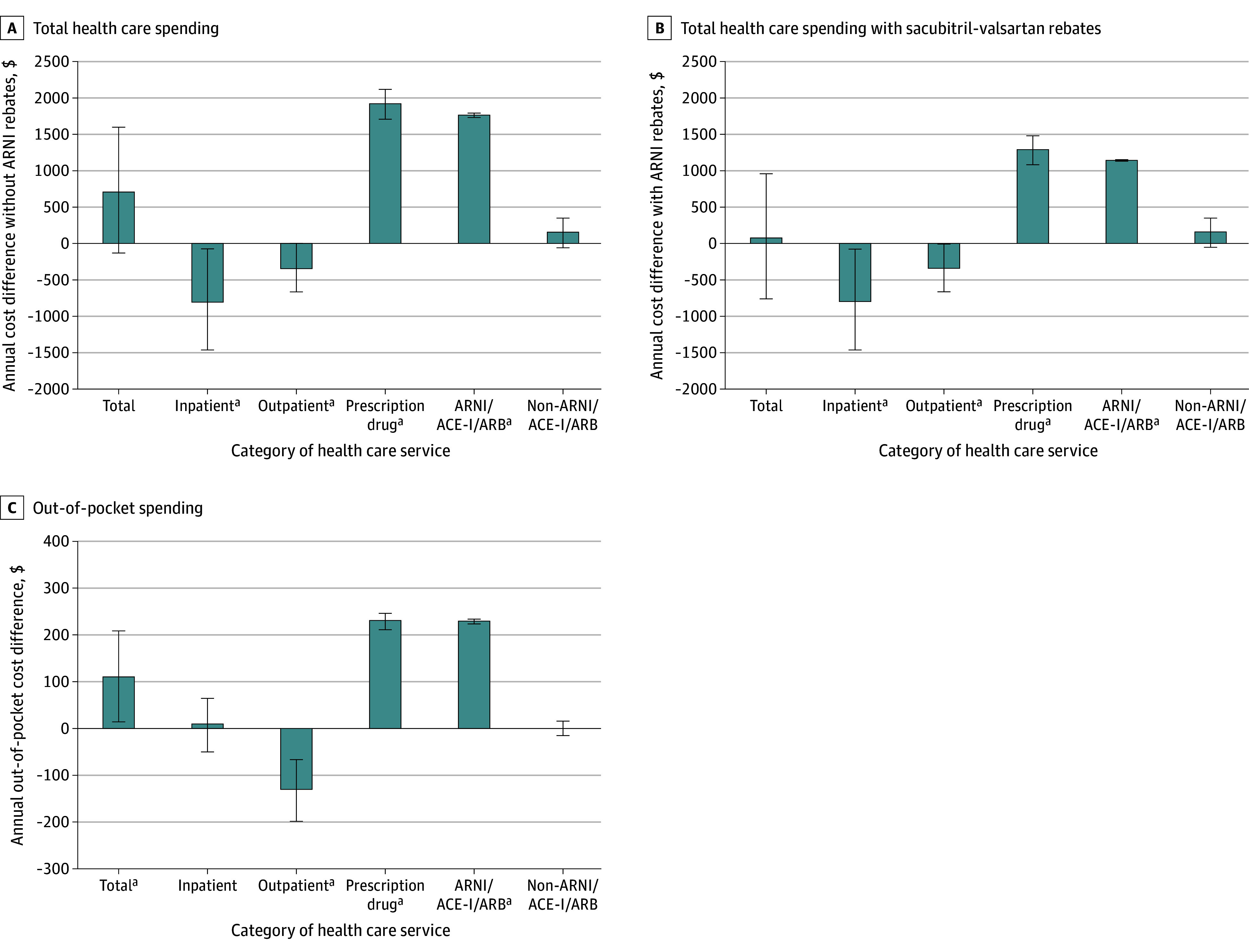
Annual Per-Person Cost Differences for the 365 Days After Initiating Sacubitril-Valsartan vs Angiotensin-Converting Enzyme Inhibitors (ACE-Is) and Angiotensin II Receptor Blockers (ARBs) ARNI indicates angiotensin receptor−neprilysin inhibitor and error bars indicate 95% CIs. ^a^Statistically significant based on 95% CIs that did not cross zero. Mean differences, mean ratios, and 95% CIs were calculated using a nonparametric bootstrapping method with 500 samples drawn with replacement.

In the primary analyses, mean (SD) annual total health care spending per person was similar for sacubitril-valsartan ($37 025 [$45 684]) and ACE-I/ARB initiators ($35 891 [$46 985]) (Table 2). The mean difference in spending per person between the 2 groups was $701 (95% CI, −$132 to $1593; Figure 2A). Compared to ACE-I/ARB initiators, sacubitril-valsartan initiators had lower mean inpatient ($17 319 [$35 605] vs $17 999 [$36 874]; difference, −$790; 95% CI, −$1468 to −$72) and outpatient ($11 376 [$17 805] vs $11 581 [$17 655]; difference, −$330; 95% CI, −$664 to −$11) spending. Mean (SD) prescription drug spending per person was $1911 (95% CI, $1704 to $2113) higher in the sacubitril-valsartan group ($6403 [$11 698]) than the ACE-I/ARB group ($4308 [$10 653]), due to higher spending on the index drugs ($1967 [$1827] vs $140 [$585]; difference, $1761; 95% CI, $1736 to $1788). This corresponded to sacubitril-valsartan initiators spending a mean of 14.2 (95% CI, 13.3 to 15.4) times more on their index medications than ACE-I/ARB initiators. There was no difference in spending on other prescription drugs between the 2 groups.

**Table 2.** Total and Out-of-Pocket Spending During the 365 Days After Initiating Sacubitril-Valsartan vs Angiotensin-Converting Enzyme Inhibitors (ACE-Is) or Angiotensin II Receptor Blockers (ARBs)

Type of cost	Initiators’ mean (SD) annual cost, $^a^		Mean (95% CI) cost, $^b^	
	Sacubitril-valsartan	ACE-I/ARBs	Difference	Ratio
Total costs without sacubitril-valsartan rebates^c^	37 025 (45 684)	35 891 (46 985)	701 (−132 to 1593)	1.02 (1.00 to 1.05)
Inpatient	17 319 (35 605)	17 999 (36 874)	−790 (−1468 to −72)	0.96 (0.92 to 1.00)
Outpatient	11 376 (17 805)	11 581 (17 655)	−330 (−664 to −11)	0.97 (0.94 to 1.00)
Prescription drug	6403 (11 698)	4308 (10 653)	1911 (1704 to 2113)	1.45 (1.39 to 1.52)
ARNI/ACE-I/ARBs	1967 (1827)	140 (585)	1761 (1736 to 1788)	14.24 (13.34 to 15.35)
Other prescription drugs^d^	4436 (11 218)	4168 (10 599)	150 (−61 to 344)	1.04 (0.99 to 1.09)
Total costs with sacubitril-valsartan rebates^c^	36 322 (45 659)	35 851 (46 973)	70 (−761 to 956)	1.00 (0.98 to 1.03)
Inpatient	17 319 (35 605)	17 999 (36 874)	−790 (−1468 to −72)	0.96 (0.92 to 1.00)
Outpatient	11 376 (17 805)	11 581 (17 655)	−330 (−664 to −11)	0.97 (0.94 to 1.00)
Prescription drug	5700 (11 494)	4268 (10 631)	1281 (1076 to 1479)	1.31 (1.25 to 1.36)
ARNI/ACE-I/ARBs	1264 (1174)	100 (374)	1130 (1114 to 1147)	12.84 (12.08 to 13.75)
Other prescription drugs^d^	4436 (11 218)	4168 (10 599)	150 (−61 to 344)	1.04 (0.99 to 1.09)
Out-of-pocket costs	4114 (4937)	3961 (5399)	109 (13 to 208)	1.03 (1.00 to 1.06)
Inpatient	1220 (2958)	1194 (3172)	9 (−51 to 64)	1.01 (0.96 to 1.05)
Outpatient	2070 (3403)	2176 (3785)	−129 (−199 to −67)	0.94 (0.91 to 0.97)
Prescription drug	825 (1063)	591 (885)	229 (211 to 246)	1.40 (1.36 to 1.43)
ARNI/ACE-I/ARBs	259 (378)	26 (102)	228 (223 to 233)	10.10 (9.44 to 10.78)
Other prescription drugs^d^	566 (869)	565 (866)	0 (−16 to 16)	1.00 (0.97 to 1.03)

Abbreviation: ARNI, angiotensin receptor−neprilysin inhibitor.

^a^ Censoring for death or disenrollment before 365 d was accounted for based on weighting by Kaplan-Meier probabilities as described by Lin et al.^22^

^b^ Mean differences, mean ratios, and 95% CIs were calculated using a nonparametric bootstrapping method with 500 samples drawn with replacement.

^c^ Total costs with and without rebates include home health services, which were not included in the inpatient, outpatient, or prescription drug costs.

^d^ All prescription drugs except sacubitril-valsartan, ACE-Is, or ARBs.

After accounting for manufacturer rebates for sacubitril-valsartan, mean (SD) annual spending per person remained similar between sacubitril-valsartan ($36 222 [$45 659]) and ACE-I/ARB ($35 851 [$46 973]) initiators (difference, $70; 95% CI, −$761 to $956; Table 2 and Figure 2B). Prescription drug spending per person averaged $1281 (95% CI, $1076 to $1479) higher in the sacubitril-valsartan group ($5700) than the ACE-I/ARB group ($4268). Mean postrebate spending on the index drugs per person was $1264 for sacubitril-valsartan initiators and $100 for ACE-I/ARB initiators (difference, $1130; 95% CI, $1114 to $1147).

### Out-of-Pocket Spending

Mean (SD) annual out-of-pocket spending per person was $109 (95% CI, $13 to $208) higher for sacubitril-valsartan initiators ($4114 [$4937]) compared to ACE-I/ARB initiators ($3961 [$5399]; Figure 2C). Mean (SD) out-of-pocket prescription drug spending per person was $229 (95% CI, $211 to $246) higher in the sacubitril-valsartan group ($825 [$1063]) than in the ACE-I/ARB group ($591 [$885]); this was associated with a $228 (95% CI, $223 to $233) higher mean out-of-pocket spending per person on sacubitril-valsartan ($259 [$378]) than on ACE-I/ARBs ($26 [$102]). Mean out-of-pocket spending on outpatient services was lower among sacubitril-valsartan than ACE-I/ARB initiators ($2070 [$3403] vs $2176 [3785]; difference, −$129; 95% CI, −$199 to −$67), and mean inpatient out-of-pocket spending was similar ($1220 [$2958] vs $1194 [3172]; difference, $9; 95% CI, −$51 to $64).

### Subgroup Analyses

In subgroup analyses, mean annual costs were higher among sacubitril-valsartan initiators than ACE-I/ARB initiators in individuals 80 years or older, those with no heart failure hospitalizations in the year prior to initiation, and those in the lowest and third quartiles of baseline health care spending; other subgroups had no significant differences in annual spending between the sacubitril-valsartan and ACE-I/ARB initiators (Table 3; eTables 5-21 in Supplement 1). Patient out-of-pocket costs were higher among sacubitril-valsartan initiators than ACE-I/ARB initiators for those 80 years or older, female patients, those with prefrailty, those without low-income subsidies, those without a heart failure hospitalization during the baseline period, and those in the 2 lowest quartiles of baseline health care costs; other subgroups had no significant differences in out-of-pocket costs between the sacubitril-valsartan and ACE-I/ARB initiators.

**Table 3.** Total and Out-of-Pocket Spending During the 365 Days After Initiating Sacubitril-Valsartan vs Angiotensin-Converting Enzyme Inhibitors (ACE-Is) or Angiotensin II Receptor Blockers (ARBs)

Type of cost	Initiators’ mean (SD) annual cost, $^a^			Mean (95% CI) cost, $^b^	
	Sacubitril-valsartan	ACE-I/ARBs		Difference	Ratio
**Total costs without sacubitril-valsartan rebates^c^**					
Age, y					
<80	38 886 (49 109)	38 217 (50 592)		−32 (−1213 to 1197)	1.00 (0.97 to 1.03)
≥80	33 773 (38 385)	31 739 (36 422)		1858 (747 to 2974)	1.06 (1.02 to 0.10)
Sex					
Female	36 194 (41 952)	35 011 (45 080)		1454 (−177 to 2848)	1.04 (0.99 to 1.09)
Male	36 923 (46 122)	35 623 (45 995)		204 (−1014 to 1290)	1.01 (0.97 to 1.04)
Race and ethnicity					
White	35 809 (43 894)	35 215 (45 848)		574 (−333 to 1520)	1.02 (0.99 to 1.04)
Other race and ethnicity^d^	41 360 (49 768)	39 581 (51 172)		833 (−1609 to 3304)	1.02 (0.96 to 1.09)
Frailty (index score)					
Not frail (<0.15)	23 417 (38 102)	22 743 (34 348)		−121 (−3400 to 2910)	1.00 (0.86 to 1.14)
Prefrail (0.15-0.24)	33 883 (42 924)	33 161 (45 193)		839 (−242 to 1914)	1.03 (0.99 to 1.06)
Frail (≥0.25)	46 491 (50 455)	45 352 (50 420)		1022 (−675 to 2901)	1.02 (0.99 to 1.07)
Low-income subsidy recipient					
Yes	43 112 (46 935)	41 188 (50 254)		1102 (−893 to 3026)	1.03 (0.98 to 1.08)
No	33 793 (44 117)	33 929 (44 753)		466 (−579 to 1412)	1.01 (0.98 to 1.04)
Heart failure hospitalization(s) during baseline period					
≥1	44 263 (48 824)	44 468 (50 201)		−1018 (−2769 to 857)	0.98 (0.94 to 1.02)
None	33 295 (42 830)	31 893 (43 774)		1489 (498 to 2590)	1.05 (1.02 to 1.09)
Baseline health care costs, by quartile					
First (lowest)	22 878 (31 071)	20 415 (28 713)		2560 (1396 to 3705)	1.13 (1.07 to 1.19)
Second	32 221 (35 874)	31 751 (39 322)		776 (−847 to 2433)	1.03 (0.97 to 1.08)
Third	40 397 (44 469)	37 746 (44 841)		1915 (70 to 3762)	1.05 (1.00 to 1.10)
Fourth (highest)	58 419 (62 017)	57 611 (59 150)		−204 (−3086 to 2705)	1.00 (0.95 to 1.05)
**Total costs with sacubitril-valsartan rebates^c^**					
Age, y					
<80	38 156 (49 094)	38 171 (50 851)		−686 (−1868 to 547)	0.98 (0.95 to 1.01)
≥80	33 112 (38339)	31 710 (36 408)		1262 (149 to 2375)	1.04 (1.00 to 1.08)
Sex					
Female	35 475 (41 910)	34 974 (45 074)		810 (−477 to 2162)	1.02 (0.99 to 1.07)
Male	36 231 (46 100)	35 581 (45 984)		−398 (−1689 to 701)	0.99 (0.95 to 1.02)
Race and ethnicity					
White	35 106 (43 868)	35 178 (45 840)		−55 (−965 to 891)	1.00 (0.97 to 1.03)
Other race and ethnicity^d^	40 638 (49 728)	39 537 (51 162)		191 (−2257 to 2656)	1.01 (0.95 to 1.07)
Frailty (index score)					
Not frail (<0.15)	22 613 (38 057)	22 685 (34 333)		−849 (−4162 to 2149)	0.96 (0.83 to 1.11)
Prefrail (0.15-0.24)	33 152 (42 901)	33 117 (45 183)		178 (−903 to 1258)	1.01 (0.97 to 1.04)
Frail (≥0.25)	45 866 (50 402)	45 324 (50 410)		482 (−1207 to 2358)	1.01 (0.97 to 1.06)
Low-income subsidy recipient					
Yes	42 259 (46 896)	41 146 (50 246)		368 (−1626 to 2276)	1.01 (0.96 to 1.06)
No	33 793 (44 117)	33 891 (44 741)		−125 (−1167 to 818)	1.00 (0.97 to 1.03)
Heart failure hospitalization(s) during baseline period					
≥1	43 625 (48 794)	44 426 (50 192)		−1578 (−3323 to 289)	0.96 (0.93 to 1.01)
None	32 562 (42 801)	31 855 (43 762)		826 (−162 to 1927)	1.03 (0.99 to 1.06)
Baseline health care costs, by quartile					
First (lowest)	22 137 (31 041)	20 380 (28 697)		1875 (717 to 3012)	1.09 (1.03 to 1.16)
Second	31 498 (35 835)	31 701 (39 311)		127 (−1493 to 1781)	1.00 (0.95 to 1.06)
Third	39 710 (44 443)	37 706 (44 831)		1199 (−925 to 3121)	1.03 (0.98 to 1.08)
Fourth (highest)	57 782 (61 950)	57 580 (59 145)		−701 (−3610 to 2154)	0.99 (0.94 to 1.04)
**Out-of-pocket costs**					
Age, y					
<80	4313 (5365)	4184 (5776)		48 (−77 to 179)	1.01 (0.98 to 1.04)
≥80	3774 (4106)	3551 (4758)		205 (69 to 334)	1.06 (1.02 to 1.10)
Sex					
Male	4142 (4830)	4027 (5441)		63 (−61 to 192)	1.02 (0.99 to 1.05)
Female	3950 (4484)	3874 (5756)		172 (10 to 339)	1.05 (1.00 to 1.09)
Race and ethnicity					
White	4081 (4707)	4000 (5500)		103 (−3 to 216)	1.03 (1.00 to 1.06)
Other race and ethnicity^d^	4132 (5770)	3935 (5174)		112 (−161 to 400)	1.03 (0.96 to 1.11)
Frailty (index score)					
Not frail (<0.15)	3178 (5244)	3013 (3803)		76 (−265 to 375)	1.03 (0.92 to 1.15)
Prefrail (0.15-0.24)	3871 (4526)	3719 (5223)		134 (37 to 244)	1.04 (1.01 to 1.07)
Frail (≥0.25)	4846 (5646)	4781 (5694)		48 (−181 to 261)	1.01 (0.96 to 1.06)
Low-income subsidy recipient					
Yes	3839 (5004)	3760 (5171)		11 (−200 to 223)	1.00 (0.95 to 1.06)
No	4202 (4854)	4074 (5524)		138 (32 to 248)	1.04 (1.01 to 1.06)
Heart failure hospitalization(s) during baseline period					
≥1	4515 (4969)	4507 (5361)		−87 (−277 to 114)	0.98 (0.94 to 1.03)
None	3906 (4897)	3730 (5157)		175 (65 to 287)	1.05 (1.02 to 1.08)
Baseline health care costs, by quartile					
First (lowest)	2861 (3217)	2562 (3201)		272 (158 to 390)	1.11 (1.06 to 1.16)
Second	3628 (3402)	3454 (3494)		174 (29 to 314)	1.05 (1.01 to 1.10)
Third	4317 (4397)	4206 (5497)		173 (−2 to 348)	1.04 (1.00 to 1.09)
Fourth (highest)	6245 (7766)	6198 (7975)		−96 (−419 to 241)	0.98 (0.93 to 1.04)

^a^ Censoring for death or disenrollment before 365 d was accounted for based on weighting by Kaplan-Meier probabilities as described by Lin et al.^22^

^b^ Mean differences, mean ratios, and 95% CIs were calculated using a nonparametric bootstrapping method with 500 samples drawn with replacement.

^c^ Total costs with and without rebates include home health services, which were not included in the inpatient, outpatient, or prescription drug costs.

^d^ Asian, Black, Hispanic, and other race or ethnicity. These data were collected from Medicare fee-for-service claims.

### Sensitivity Analyses

The results of our analysis using an as-treated approach are shown in eTable 22 in Supplement 1. Only 4400 patients (16.0%; eTable 23 in Supplement 1) completed 365 days of follow-up; 48.0% were censored due to discontinuing their index sacubitril-valsartan or ACE-I/ARB use and 4.5%, for switching to a medication in the opposite group. The mean (SD) annual total spending per person was $1796 (95% CI, $1309 to $2346) lower in sacubitril-valsartan initiators ($21 452 [$28 810]) than in ACE-I/ARB initiators ($22 375 [$31 007]), and mean out-of-pocket spending was $156 (95% CI, $107 to $212) lower in the sacubitril-valsartan group ($2342 [$3258]) than in the ACE-I/ARB group ($2428 [$3827]). The higher prescription drug costs among sacubitril-valsartan initiators were offset by lower mean inpatient ($8802 [$19 891] vs $10 414 [$22 630]; difference, −$1699; 95% CI, −$2060 to −$1351) and outpatient ($7131 [$13 277] vs $7737 [$13 758]; difference −$682; 95% CI, −$903 to −$470) spending. In additional sensitivity analyses, repeating the main intention-to-treat analyses using 1000, rather than 500, bootstraps yielded nearly identical results (eTable 24 in Supplement 1).

## Discussion

In this cohort study of Medicare beneficiaries with HFrEF, those initiating sacubitril-valsartan and ACE-I/ARBs had similar total health care costs in the subsequent year. Higher total spending on sacubitril-valsartan was offset by lower inpatient and outpatient costs. However, sacubitril-valsartan initiators experienced higher out-of-pocket costs than those initiating ACE-I/ARBs, which can have important implications for affordability and equitable access to this newer treatment option.

Despite its high price, sacubitril-valsartan has previously been found to be a cost-effective treatment for heart failure as compared to ACE-I/ARBs.^14,28,29,30,31,32^ Although we did not compare clinical outcomes to estimate cost-effectiveness, our study findings align with those showing that the higher cost of sacubitril-valsartan was offset by lower spending on other health care services, even over a short period of follow-up. However, despite the high cost of sacubitril-valsartan that was offset by lower total spending on inpatient and outpatient care, out-of-pocket spending remained higher among patients using sacubitril-valsartan. For other chronic health conditions, high out-of-pocket spending is associated with lower adherence, and in some cases, worse clinical outcomes.^33^ Although our study did not directly compare medication adherence between sacubitril-valsartan and ACE-I/ARB initiators, total spending was lower for sacubitril-valsartan initiators in the as-treated analyses than in the intention-to-treat analyses, which suggests that cost-related nonadherence to sacubitril-valsartan may offset the drug’s benefits associated with less spending on heart failure complications (eg, hospitalizations). Another study^34^ found that patients who initiated sacubitril-valsartan had lower adherence at 6 months compared to those receiving other heart failure treatment regimens.

The findings of our subgroup analyses suggest that the high cost of sacubitril-valsartan was not offset by associated reductions in inpatient and outpatient spending among Medicare beneficiaries 80 years or older, without recent heart failure hospitalizations, or with lower baseline health care spending. Older individuals with more severe heart failure were not well represented in the PARADIGM-HF trial^11^; participants were mostly younger (mean age, 63.8 years) and only one-quarter had New York Heart Association class III or IV heart failure. Although there was no signal in the trial toward lower efficacy by age or heart failure severity, our findings in the routine clinical setting suggest that the cost effects of sacubitril-valsartan may differ by age and illness severity. They also highlight the importance of better understanding the absolute benefits, risks, and costs of this medication within these subgroups, which can help inform shared decision-making between clinicians and patients. Future studies may offer valuable insights into the differential cost effects of sacubitril-valsartan among various subgroups.

The high cost of sacubitril-valsartan has received substantial attention from policymakers. In August 2023, it was selected as one of the first 10 drugs to undergo Medicare price negotiation under the Inflation Reduction Act.^35^ Claims-based spending data can inform these types of negotiations at the federal and state levels. Medicare’s negotiation of a lower price could lead to an even more favorable cost profile for sacubitril-valsartan. It will be important to evaluate whether this results in lower out-of-pocket costs, given that these expenses create financial challenges for many patients, especially those with lower incomes and who reside in rural communities.^36,37^

### Limitations

Our study has limitations. First, we included only Medicare fee-for-service beneficiaries, so the results may not be generalizable to those in Medicare Advantage plans or younger patients with heart failure who have private insurance or Medicaid. Second, the Kaplan-Meier−based adjustment for incomplete follow-up assumes noninformative censoring; this could produce biased results if costs differed among those censored before 365 days compared to those with complete follow-up. Third, total and out-of-pocket spending were based on insurance claims data, and thus, excluded indirect costs (eg, caregiver loss of productivity) and health care services that were not reimbursed by Medicare (eg, over-the-counter medications). Fourth, we were unable to reliably stratify health care spending into cardiac vs noncardiac expenditures. Fifth, we may have overestimated actual out-of-pocket spending because we could not account for patient assistance programs that may have offset these costs for some patients. Sixth, we did not account for how the results may have been affected by recalls of several ARB formulations during the study period, although prior research suggests that these recalls did not meaningfully disrupt ARB use or adherence.^38^ Seventh, we only included patients initiating sacubitril-valsartan de novo in a new user cohort design and did not consider those who switched from an ACE-I/ARB to sacubitril-valsartan. Therefore, our results may not generalize to all sacubitril-valsartan users. Lastly, as with all observational studies, the analyses may be influenced by potential residual confounding.

## Conclusions

This cohort study found that among Medicare beneficiaries with heart failure, annual total health care spending was similar after initiating sacubitril-valsartan or an ACE-I/ARB, but sacubitril-valsartan recipients experienced higher out-of-pocket costs. These routine spending patterns for sacubitril-valsartan have important clinical implications given that even when the high cost of sacubitril-valsartan is offset by lower inpatient and outpatient spending, the greater cost burden on Medicare beneficiaries may contribute to wider health disparities by limiting access and adherence to sacubitril-valsartan.

## Notes

Supplement 1. eTable 1. Medicare files used to derive total and out-of-pocket spending during the 365 days following ARNI or ACE-I/ARB initiation eTable 2. Complete list of unmatched baseline characteristics, October 1, 2016 to December 31, 2019 eTable 3. Complete list of propensity-score matched baseline characteristics, October 1, 2016 to December 31, 2019 eTable 4. Censoring reasons for our 13,775 matched pairs in our intention-to-treat primary analyses eTable 5. Total health care and out-of-pocket costs during the 365 days following sacubitril-valsartan or ARNI/ACE-I/ARBs initiation using an intention-to-treat approach for patients less than 80 years old eTable 6. Total health care and out-of-pocket costs during the 365 days following sacubitril-valsartan or ARNI/ACE-I/ARBs initiation using an intention-to-treat approach for patients greater than or equal to 80 years old eTable 7. Total health care and out-of-pocket costs during the 365 days following sacubitril-valsartan or ARNI/ACE-I/ARBs initiation using an intention-to-treat approach for patients with reported male sex eTable 8. Total health care and out-of-pocket costs during the 365 days following sacubitril-valsartan or ARNI/ACE-I/ARBs initiation using an intention-to-treat approach for patients with reported female sex eTable 9. Total health care and out-of-pocket costs during the 365 days following sacubitril-valsartan or ARNI/ACE-I/ARBs initiation using an intention-to-treat approach for patients who reported White race eTable 10. Total health care and out-of-pocket costs during the 365 days following sacubitril-valsartan or ARNI/ACE-I/ARBs initiation using an intention-to-treat approach for patients who reported Asian, Black, Hispanic, or other race eTable 11. Total health care and out-of-pocket costs during the 365 days following sacubitril-valsartan or ARNI/ACE-I/ARBs initiation using an intention-to-treat approach for patients with low frailty scores (frailty score < 0.15) eTable 12. Total health care and out-of-pocket costs during the 365 days following sacubitril-valsartan or ARNI/ACE-I/ARBs initiation using an intention-to-treat approach for patients with moderate frailty scores (0.15 ≤ frailty score < 0.25) eTable 13. Total health care and out-of-pocket costs during the 365 days following sacubitril-valsartan or ARNI/ACE-I/ARBs initiation using an intention-to-treat approach for patients with high frailty scores (frailty score ≥ 0.25) eTable 14. Total health care and out-of-pocket costs during the 365 days following sacubitril-valsartan or ARNI/ACE-I/ARBs initiation using an intention-to-treat approach for patients who are low-income subsidy recipients eTable 15. Total health care and out-of-pocket costs during the 365 days following sacubitril-valsartan or ARNI/ACE-I/ARBs initiation using an intention-to-treat approach for patients who are not low-income subsidy recipients eTable 16. Total health care and out-of-pocket costs during the 365 days following sacubitril-valsartan or ARNI/ACE-I/ARBs initiation using an intention-to-treat approach for patients with at least one heart failure hospitalization during the baseline period eTable 17. Total health care and out-of-pocket costs during the 365 days following sacubitril-valsartan or ARNI/ACE-I/ARBs initiation using an intention-to-treat approach for patients with no heart failure hospitalization during the baseline period eTable 18. Total health care and out-of-pocket costs during the 365 days following sacubitril-valsartan or ARNI/ACE-I/ARBs initiation using an intention-to-treat approach for patients with baseline health care costs in the lowest quartile eTable 19. Total health care and out-of-pocket costs during the 365 days following sacubitril-valsartan or ARNI/ACE-I/ARBs initiation using an intention-to-treat approach for patients with baseline health care costs in the second lowest quartile eTable 20. Total health care and out-of-pocket costs during the 365 days following sacubitril-valsartan or ARNI/ACE-I/ARBs initiation using an intention-to-treat approach for patients with baseline health care costs in the second highest quartile eTable 21. Total health care and out-of-pocket costs during the 365 days following sacubitril-valsartan or ARNI/ACE-I/ARBs initiation using an intention-to-treat approach for patients with baseline health care costs in the highest quartile eTable 22. Total health care and out-of-pocket costs during the 365 days following sacubitril-valsartan or ARNI/ACE-I/ARBs initiation using an as-treated approach as sensitivity analyses eTable 23. Censoring reasons for our 13,775 matched pairs in our as-treated sensitivity analyses eTable 24. Total and out-of-pocket spending during the 365 days following sacubitril-valsartan or ARNI/ACE-I/ARBs initiation. Sensitivity analyses using 1,000 bootstraps, compared to using 500 bootstraps. eFigure 1. Propensity scores by exposure group for all patients prior to matching eFigure 2. Propensity scores by exposure group for the matched cohort

Supplement 2. Data Sharing Statement
