# Supplementary material for: Health Care Spending After Initiating Sacubitril-Valsartan vs Renin-Angiotensin System Blockers for Heart Failure Treatment
Source: JAMA Health Forum. 2025 Feb 14;6(2):e245385. doi: 10.1001/jamahealthforum.2024.5385 (PMC11829231; doi:10.1001/jamahealthforum.2024.5385)
Supplement: Supplement 1. — eTable 1. Medicare files used to derive total and out-of-pocket spending during the 365 days following ARNI or ACE-I/ARB initiation eTable 2. Complete list of unmatched baseline characteristics, October 1, 2016 to December 31, 2019 eTable 3. Complete list of propensity-score matched baseline characteristics, October 1, 2016 to December 31, 2019 eTable 4. Censoring reasons for our 13,775 matched pairs in our intention-to-treat primary analyses eTable 5. Total health care and out-of-pocket costs during the 365 days following sacubitril-valsartan or ARNI/ACE-I/ARBs initiation using an intention-to-treat approach for patients less than 80 years old eTable 6. Total health care and out-of-pocket costs during the 365 days following sacubitril-valsartan or ARNI/ACE-I/ARBs initiation using an intention-to-treat approach for patients greater than or equal to 80 years old eTable 7. Total health care and out-of-pocket costs during the 365 days following sacubitril-valsartan or ARNI/ACE-I/ARBs initiation using an intention-to-treat approach for patients with reported male sex eTable 8. Total health care and out-of-pocket costs during the 365 days following sacubitril-valsartan or ARNI/ACE-I/ARBs initiation using an intention-to-treat approach for patients with reported female sex eTable 9. Total health care and out-of-pocket costs during the 365 days following sacubitril-valsartan or ARNI/ACE-I/ARBs initiation using an intention-to-treat approach for patients who reported White race eTable 10. Total health care and out-of-pocket costs during the 365 days following sacubitril-valsartan or ARNI/ACE-I/ARBs initiation using an intention-to-treat approach for patients who reported Asian, Black, Hispanic, or other race eTable 11. Total health care and out-of-pocket costs during the 365 days following sacubitril-valsartan or ARNI/ACE-I/ARBs initiation using an intention-to-treat approach for patients with low frailty scores (frailty score < 0.15) eTable 12. Total health care and out-of [file jamahealthforum-e245385-s001.pdf]

Supplemental Online Content

Hwang CS, Desai RJ, Kesselheim AS, Levin R, Kattinakere Sreedhara S, Rome BN. Health Care Spending After Initiating Sacubitril-Valsartan vs Renin-Angiotensin System Blockers for Heart Failure Treatment. *JAMA Health Forum*. 2025;6(2):e245385. doi:10.1001/jamahealthforum.2024.5385

eTable 1. Medicare files used to derive total and out-of-pocket spending during the 365 days following ARNI or ACE-I/ARB initiation

eTable 2. Complete list of unmatched baseline characteristics, October 1, 2016 to December 31, 2019

eTable 3. Complete list of propensity-score matched baseline characteristics, October 1, 2016 to December 31, 2019

eTable 4. Censoring reasons for our 13,775 matched pairs in our intention-to-treat primary analyses

eTable 5. Total health care and out-of-pocket costs during the 365 days following sacubitril-valsartan or ARNI/ACE-I/ARBs initiation using an intention-to-treat approach for patients less than 80 years old

eTable 6. Total health care and out-of-pocket costs during the 365 days following sacubitril-valsartan or ARNI/ACE-I/ARBs initiation using an intention-to-treat approach for patients greater than or equal to 80 years old

eTable 7. Total health care and out-of-pocket costs during the 365 days following sacubitril-valsartan or ARNI/ACE-I/ARBs initiation using an intention-to-treat approach for patients with reported male sex

eTable 8. Total health care and out-of-pocket costs during the 365 days following sacubitril-valsartan or ARNI/ACE-I/ARBs initiation using an intention-to-treat approach for patients with reported female sex

eTable 9. Total health care and out-of-pocket costs during the 365 days following sacubitril-valsartan or ARNI/ACE-I/ARBs initiation using an intention-to-treat approach for patients who reported White race

eTable 10. Total health care and out-of-pocket costs during the 365 days following sacubitril-valsartan or ARNI/ACE-I/ARBs initiation using an intention-to-treat approach for patients who reported Asian, Black, Hispanic, or other race

eTable 11. Total health care and out-of-pocket costs during the 365 days following sacubitril-valsartan or ARNI/ACE-I/ARBs initiation using an intention-to-treat approach for patients with low frailty scores (frailty score < 0.15)

eTable 12. Total health care and out-of-pocket costs during the 365 days following sacubitril-valsartan or ARNI/ACE-I/ARBs initiation using an intention-to-treat approach for patients with moderate frailty scores (0.15 ≤ frailty score < 0.25)

eTable 13. Total health care and out-of-pocket costs during the 365 days following sacubitril-valsartan or ARNI/ACE-I/ARBs initiation using an intention-to-treat approach for patients with high frailty scores (frailty score ≥ 0.25)

© 2025 Hwang CS et al. *JAMA Health Forum*

eTable 14. Total health care and out-of-pocket costs during the 365 days following sacubitril-valsartan or ARNI/ACE-I/ARBs initiation using an intention-to-treat approach for patients who are low-income subsidy recipients

eTable 15. Total health care and out-of-pocket costs during the 365 days following sacubitril-valsartan or ARNI/ACE-I/ARBs initiation using an intention-to-treat approach for patients who are not low-income subsidy recipients

eTable 16. Total health care and out-of-pocket costs during the 365 days following sacubitril-valsartan or ARNI/ACE-I/ARBs initiation using an intention-to-treat approach for patients with at least one heart failure hospitalization during the baseline period

eTable 17. Total health care and out-of-pocket costs during the 365 days following sacubitril-valsartan or ARNI/ACE-I/ARBs initiation using an intention-to-treat approach for patients with no heart failure hospitalization during the baseline period

eTable 18. Total health care and out-of-pocket costs during the 365 days following sacubitril-valsartan or ARNI/ACE-I/ARBs initiation using an intention-to-treat approach for patients with baseline health care costs in the lowest quartile

eTable 19. Total health care and out-of-pocket costs during the 365 days following sacubitril-valsartan or ARNI/ACE-I/ARBs initiation using an intention-to-treat approach for patients with baseline health care costs in the second lowest quartile

eTable 20. Total health care and out-of-pocket costs during the 365 days following sacubitril-valsartan or ARNI/ACE-I/ARBs initiation using an intention-to-treat approach for patients with baseline health care costs in the second highest quartile

eTable 21. Total health care and out-of-pocket costs during the 365 days following sacubitril-valsartan or ARNI/ACE-I/ARBs initiation using an intention-to-treat approach for patients with baseline health care costs in the highest quartile

eTable 22. Total health care and out-of-pocket costs during the 365 days following sacubitril-valsartan or ARNI/ACE-I/ARBs initiation using an as-treated approach as sensitivity analyses

eTable 23. Censoring reasons for our 13,775 matched pairs in our as-treated sensitivity analyses

eTable 24. Total and out-of-pocket spending during the 365 days following sacubitril-valsartan or ARNI/ACE-I/ARBs initiation. Sensitivity analyses using 1,000 bootstraps, compared to using 500 bootstraps

eFigure 1. Propensity scores by exposure group for all patients prior to matching

eFigure 2. Propensity scores by exposure group for the matched cohort

This supplemental material has been provided by the authors to give readers additional information about their work.

© 2025 Hwang CS et al. *JAMA Health Forum*

eTable 1. Medicare files used to derive total and out-of-pocket spending during the 365 days following ARNI or ACE-I/ARB initiation.

**Outcome Measure**	**Variables**
**Total costs**	Sum of total inpatient, outpatient, and prescription drug costs
Inpatient costs	Inpatient base file variables:<br>CLM_PMT_AMT + (CLM_PASS_THRU_PER_DIEM_AMT *CLM_UTLZTN_DAY_CNT) + NCH_BENE_IP_DDCTBL_AMT + NCH_BENE_PTA_COINSRNC_LBLTY_AM + NCH_BENE_BLOOD_DDCTBL_LBLTY_AM + NCH_PRMRY_PYR_CLM_PD_AMT<br>+<br>SNF file variables:<br>CLM_PMT_AMT + NCH_BENE_IP_DDCTBL_AMT + NCH_BENE_PTA_COINSRNC_LBLTY_AM + NCH_BENE_BLOOD_DDCTBL_LBLTY_AM + NCH_PRMRY_PYR_CLM_PD_AMT
Outpatient costs	Outpatient base file variables:<br>CLM_PMT_AMT + NCH_BENE_PTB_DDCTBL_AMT + NCH_BENE_PTB_COINSRNC_AMT + NCH_BENE_BLOOD_DDCTBL_LBLTY_AM + NCH_PRMRY_PYR_CLM_PD_AMT<br>+<br>Carrier claims line file variables:<br>LINE_NCH_PMT_AMT + LINE_BENE_PRMRY_PYR_PD_AMT + (LINE_ALOWD_CHRG_AMT - LINE_NCH_PMT_AMT - LINE_BENE_PRMRY_PYR_PD_AMT) + LINE_BENE_PRMRY_PYR_PD_AMT<br>+<br>Durable medical equipment line file variables:<br>LINE_NCH_PMT_AMT + LINE_BENE_PRMRY_PYR_PD_AMT + (LINE_ALOWD_CHRG_AMT - LINE_NCH_PMT_AMT - LINE_BENE_PRMRY_PYR_PD_AMT) + LINE_BENE_PRMRY_PYR_PD_AMT
Prescription drug costs	Part D event file variables:<br>PTNT_PAY_AMT + LICS_AMT + OTHR_TROOP_AMT + PLRO_AMT + CVRD_D_PLAN_PD_AMT + NCVRD_PLAN_PD_AMT
**Total out-of-pocket costs**	Sum of out-of-pocket inpatient, outpatient, and prescription drug costs
Inpatient costs	Inpatient base file variables:<br>NCH_BENE_IP_DDCTBL_AMT + NCH_BENE_PTA_COINSRNC_LBLTY_AM + NCH_BENE_BLOOD_DDCTBL_LBLTY_AM + NCH_PRMRY_PYR_CLM_PD_AMT<br>+<br>SNF file variables:<br>NCH_BENE_IP_DDCTBL_AMT + NCH_BENE_PTA_COINSRNC_LBLTY_AM + NCH_BENE_BLOOD_DDCTBL_LBLTY_AM
Outpatient costs	Outpatient base file variables:<br>NCH_BENE_PTB_DDCTBL_AMT + NCH_BENE_PTB_COINSRNC_AMT + NCH_BENE_BLOOD_DDCTBL_LBLTY_AM<br>+

© 2025 Hwang CS et al. *JAMA Health Forum*

	Carrier claims line file variables:<br>LINE_ALOWD_CHRG_AMT - LINE_NCH_PMT_AMT - LINE_BENE_PRMRY_PYR_PD_AMT<br>+<br>Durable medical equipment line file variables:<br>LINE_ALOWD_CHRG_AMT - LINE_NCH_PMT_AMT - LINE_BENE_PRMRY_PYR_PD_AMT
Prescription drug costs	Part D event file variables:<br>PTNT_PAY_AMT

© 2025 Hwang CS et al. *JAMA Health Forum*

eTable 2. Complete list of *unmatched* baseline characteristics, October 1, 2016 to December 31, 2019.

Variable	Sacubitril-Valsartan Initiators, N = 15,085	ACE-I/ARB Initiators, N = 28,906	Standardized Difference
**Demographics, N (%)**			
Age			
65-69 years	2428 (16.1)	5545 (19.2)	-0.0811
70-74 years	3305 (21.9)	6624 (22.9)	-0.0242
75-79 years	3426 (22.7)	6314 (21.8)	0.0209
80-84 years	2934 (19.4)	5144 (17.8)	0.0425
85-89 years	1952 (12.9)	3457 (12.0)	0.0297
90 years or older	1040 (6.9)	1822 (6.3)	0.0238
Male sex	9639 (63.9)	18327 (63.4)	0.0103
Race/ethnicity			
White	12707 (84.2)	23450 (81.1)	0.0823
Asian	240 (1.6)	430 (1.5)	0.0084
Black	1465 (9.7)	3589 (12.4)	-0.0863
Hispanic	257 (1.7)	542 (1.9)	-0.0129
Other	53 (0.4)	161 (0.6)	-0.0307
Unknown	363 (2.4)	734 (2.5)	-0.0086
Census region			
Midwest	2931 (19.4)	6937 (24.0)	-0.1110
Northeast	2701 (17.9)	5845 (20.2)	-0.0590
South	7261 (48.1)	11422 (39.5)	0.1744
West	2192 (14.5)	4702 (16.3)	-0.0481
Low-income subsidy	4200 (27.8)	8711 (30.1)	-0.0506
Year of cohort entry			
2016, quarter 4	2377 (8.2)	605 (4.0)	-0.1764
2017, quarter 1	2694 (9.3)	836 (5.5)	-0.1444
2017, quarter 2	2355 (8.1)	990 (6.6)	-0.0607
2017, quarter 3	2085 (7.2)	960 (6.4)	-0.0338
2017, quarter 4	2102 (7.3)	910 (6.0)	-0.0498
2018, quarter 1	2706 (9.4)	1283 (8.5)	-0.0300
2018, quarter 2	2343 (8.1)	1231 (8.2)	0.0020
2018, quarter 3	2000 (6.9)	1131 (7.5)	0.0224
2018, quarter 4	1972 (6.8)	1132 (7.5)	0.0265
2019, quarter 1	2384 (8.2)	1599 (10.6)	0.0806
2019, quarter 2	2125 (7.4)	1502 (10.0)	0.0928
2019, quarter 3	1860 (6.4)	1387 (9.2)	0.1030
2019, quarter 4	1903 (6.6)	1519 (10.1)	0.1265
**Comorbidities, N (%)**			
Acute kidney injury	4877 (32.3)	11180 (38.7)	-0.1329
Anemia	7706 (51.1)	15318 (53.0)	-0.038
Atrial fibrillation	9462 (62.7)	17919 (62.0)	0.0152
Cancer	2979 (19.7)	6158 (21.3)	-0.0385
Cardiac resynchronization therapy	1672 (11.1)	3427 (11.9)	-0.0242
Chronic kidney disease	6998 (46.4)	13290 (46.0)	0.0083
Chronic obstructive pulmonary disease	6430 (42.6)	12749 (44.1)	-0.0299
Coronary artery disease	4317 (28.6)	8018 (27.7)	0.0196
Coronary revascularization (stent or CABG)	1347 (8.9)	3648 (12.6)	-0.1193
Defibrillation	5750 (38.1)	9717 (33.6)	0.0940
Dementia	1744 (11.6)	4428 (15.3)	-0.1104
Depression	3933 (26.1)	8815 (30.5)	-0.0983

© 2025 Hwang CS et al. *JAMA Health Forum*

Diabetes	8979 (59.5)	17820 (61.6)	-0.0435
Fractures	1777 (11.8)	4183 (14.5)	-0.0798
Hyperkalemia	2020 (13.4)	4702 (16.3)	-0.0810
Hypertension	14423 (95.6)	27620 (95.6)	0.0030
Hypotension	3443 (22.8)	7826 (27.1)	-0.0983
Obesity	5154 (34.2)	9040 (31.3)	0.0617
Other arrhythmias (excluding atrial fibrillation)	8837 (58.6)	19241 (66.6)	-0.1655
Peripheral vascular disease	10044 (66.6)	18778 (65.0)	0.0342
Pneumonia	3856 (25.6)	9561 (33.1)	-0.1656
Psychosis or delirium	367 (2.4)	1076 (3.7)	-0.0747
Pulmonary hypertension	605 (4.0)	1106 (3.8)	0.0095
Sleep apnea	3966 (26.3)	6793 (23.5)	0.0646
Smoking	7412 (49.1)	16413 (56.8)	-0.1536
Stroke or transient ischemic attack	2107 (14.0)	4977 (17.2)	-0.0897
Valvular disorders	3414 (22.6)	6282 (21.7)	0.0216
Deep vein thrombosis or pulmonary embolism	1206 (8.0)	2936 (10.2)	-0.0753
**Medications, N (%)**			
Cardiac medications			
Beta blockers	13275 (88.0)	26420 (91.4)	-0.1120
Calcium channel blockers	3333 (22.1)	7844 (27.1)	-0.1172
Digoxin	2326 (15.4)	4307 (14.9)	0.0145
Hydralazine			
Loop diuretics	11961 (79.3)	21588 (74.7)	0.1096
Nitrates	2610 (17.3)	4980 (17.2)	0.0020
Other diuretics (except loop and thiazide diuretics)	4953 (32.8)	8222 (28.4)	0.0953
Other lipid lower therapies (excluding statins)	1585 (10.5)	2338 (8.1)	0.0834
Statins	10399 (68.9)	20332 (70.3)	-0.0305
Sodium-glucose cotransporter-2 (SGLT2) inhibitors	308 (2.0)	342 (1.2)	0.0682
Thiazide diuretics	2106 (14.0)	2992 (10.4)	0.1106
Diabetes medications			
Dipeptidyl peptidase 4 (DPP-4) inhibitors	1203 (8.0)	1934 (6.7)	0.0493
Glucagon-like peptide 1 (GLP-1) agonists	383 (2.5)	458 (1.6)	0.0673
Insulin	2764 (18.3)	5561 (19.2)	-0.0234
Metformin	2435 (16.1)	5316 (18.4)	-0.0595
Sulfonylureas	1863 (12.4)	3849 (13.3)	-0.0289
Other medications			
Anti-arrhythmics	3093 (20.5)	5752 (19.9)	0.0151
Anti-coagulants	7004 (46.4)	12807 (44.3)	0.0427
Anti-platelets	4279 (28.4)	7952 (27.5)	0.0191
Non-steroidal anti-inflammatory drugs (NSAIDs)	2564 (17.0)	4668 (16.1)	0.0228
Opioids	5899 (39.1)	11953 (41.4)	-0.0458
**Burden of comorbidities, mean (SD)**			
Combined comorbidity score	7.227 (3.224)	7.847 (3.423)	-0.1863
Frailty score	0.219 (0.055)	0.228 (0.06)	-0.1652
**Health care utilization within past 365 days**			
No. emergency department visits, mean (SD)	2.136 (2.675)	2.931 (3.537)	-0.2536
No. hospitalizations, mean (SD)			
Heart failure in primary diagnosis position	0.447 (0.859)	0.618 (1.034)	-0.1797
Heart failure NOT in primary diagnosis position	0.91 (1.356)	1.377 (1.678)	-0.3056
No. office visits, mean (SD)			
Cardiology	10.614 (10.8)	10.875 (11.336)	-0.0237
Internal medicine or family medicine	18.446 (26.914)	24.948 (36.154)	-0.2040
No. dispensed medications, mean (SD)			

© 2025 Hwang CS et al. *JAMA Health Forum*

Brand-name medications	4.276 (3.128)	4.007 (3.098)	0.0864
Generic medications	10.351 (4.902)	9.634 (5.010)	0.1446
Laboratory tests, imaging, and vaccination, N (%)			
Hemoglobin A1c	8915 (59.1)	16665 (57.7)	0.0293
Colon cancer screening	2039 (13.5)	3878 (13.4)	0.0030
Creatinine	14711 (97.5)	27713 (95.9)	0.0923
Echocardiography	13320 (88.3)	26260 (90.8)	-0.0834
Electrocardiography	13812 (91.6)	27307 (94.5)	-0.1142
Influenza vaccination	9119 (60.5)	17001 (58.8)	0.0334
Mammography	1072 (7.1)	2054 (7.1)	0.0000
Microalbuminuria	3069 (20.3)	5980 (20.7)	-0.0085
Pneumococcal vaccination	2476 (16.4)	5166 (17.9)	-0.0387
Prostate cancer screening	3822 (25.3)	6282 (21.7)	0.0850
Shingles vaccination	497 (3.3)	735 (2.5)	0.0447
**Health care costs within past 365 days, mean (SD)**			
Log transformed total cost*	10.182 (1.045)	10.438 (1.1)	-0.2383
Total cost	43398 (47313)	56553 (60127)	-0.2432

*Log-transformed to better represent the right-skewed distribution of cost data.

© 2025 Hwang CS et al. *JAMA Health Forum*

eTable 3. Complete list of propensity-score *matched* baseline characteristics, October 1, 2016 to December 31, 2019.

Variable	Sacubitril-Valsartan Initiators, N = 13,755	ACE-I/ARB Initiators, N = 13,755 patients	Standardized Difference
**Demographics, N (%)**			
Age			
65-69 years	2264 (16.5)	2343 (17.0)	-0.0154
70-74 years	3058 (22.2)	2996 (21.8)	0.0109
75-79 years	3115 (22.6)	3018 (21.9)	0.0169
80-84 years	2633 (19.1)	2665 (19.4)	-0.0059
85-89 years	1762 (12.8)	1802 (13.1)	-0.0087
90 years or older	923 (6.7)	931 (6.8)	-0.0023
Male sex	8770 (63.8)	8791 (63.9)	-0.0031
Race/ethnicity			
White	11521 (83.8)	11563 (84.1)	-0.0083
Asian	220 (1.6)	226 (1.6)	-0.0035
Black	1394 (10.1)	1353 (9.8)	0.0099
Hispanic	235 (1.7)	229 (1.7)	0.0033
Other	51 (0.4)	55 (0.4)	-0.0047
Unknown	334 (2.4)	329 (2.4)	0.0023
Census region			
Midwest	2798 (20.3)	2789 (20.3)	0.0016
Northeast	2537 (18.4)	2521 (18.3)	0.0030
South	6355 (46.2)	6373 (46.3)	-0.0026
West	2065 (15.0)	2072 (15.1)	-0.0014
Low-income subsidy	3784 (27.5)	3704 (26.9)	0.01308
Year of cohort entry			
2016, quarter 4	601 (4.4)	632 (4.6)	-0.0109
2017, quarter 1	823 (6.0)	770 (5.6)	0.0165
2017, quarter 2	952 (6.9)	950 (6.9)	0.0006
2017, quarter 3	923 (6.7)	883 (6.4)	0.0118
2017, quarter 4	872 (6.3)	903 (6.6)	-0.0092
2018, quarter 1	1202 (8.7)	1195 (8.7)	0.0018
2018, quarter 2	1149 (8.4)	1144 (8.3)	0.0013
2018, quarter 3	1043 (7.6)	1022 (7.4)	0.0058
2018, quarter 4	1031 (7.5)	1058 (7.7)	-0.0074
2019, quarter 1	1407 (10.2)	1448 (10.5)	-0.0098
2019, quarter 2	1304 (9.5)	1290 (9.4)	0.0035
2019, quarter 3	1177 (8.6)	1212 (8.8)	-0.0090
2019, quarter 4	1271 (9.2)	1248 (9.1)	0.0058
**Comorbidities, N (%)**			
Acute kidney injury	4578 (33.3)	4599 (33.4)	-0.0032
Anemia	7012 (51.0)	7066 (51.4)	-0.0078
Atrial fibrillation	8650 (62.9)	8684 (63.1)	-0.0051
Cancer	2769 (20.1)	2787 (20.3)	-0.0033
Cardiac resynchronization therapy	1580 (11.5)	1597 (11.6)	-0.0038
Chronic kidney disease	6329 (46.0)	6369 (46.3)	-0.0058
Chronic obstructive pulmonary disease	5849 (42.5)	5875 (42.7)	-0.0038
Coronary artery disease	3869 (28.1)	3869 (28.1)	0.0000
Coronary revascularization (stent or CABG)	1304 (9.5)	1289 (9.4)	0.0037
Defibrillation	5191 (37.7)	5267 (38.3)	-0.0114
Dementia	1640 (11.9)	1664 (12.1)	-0.0054
Depression	3670 (26.7)	3699 (26.9)	-0.0048
Diabetes	8188 (59.5)	8176 (59.4)	0.0018

© 2025 Hwang CS et al. *JAMA Health Forum*

Fractures	1666 (12.1)	1713 (12.5)	-0.0104
Hyperkalemia	1854 (13.5)	1893 (13.8)	-0.0083
Hypertension	13144 (95.6)	13145 (95.6)	-0.0003
Hypotension	3210 (23.3)	3252 (23.6)	-0.0072
Obesity	4615 (33.6)	4632 (33.7)	-0.0026
Other arrhythmias (excluding atrial fibrillation)	8289 (60.3)	8239 (59.9)	0.0074
Peripheral vascular disease	9133 (66.4)	9152 (66.5)	-0.0029
Pneumonia	3658 (26.6)	3677 (26.7)	-0.0031
Psychosis or delirium	348 (2.5)	358 (2.6)	-0.0046
Pulmonary hypertension	551 (4.0)	545 (4.0)	0.0023
Sleep apnea	3545 (25.8)	3596 (26.1)	-0.0085
Smoking	6952 (50.5)	6987 (50.8)	-0.0051
Stroke or transient ischemic attack	1955 (14.2)	1983 (14.4)	-0.0058
Valvular disorders	3082 (22.4)	3141 (22.8)	-0.0103
Deep vein thrombosis or pulmonary embolism	1146 (8.3)	1157 (8.4)	-0.0029
**Medications, N (%)**			
Cardiac medications			
Beta blockers	12241 (89.0)	12241 (89.0)	0.0000
Calcium channel blockers	3143 (22.8)	3122 (22.7)	0.0036
Digoxin	2132 (15.5)	2118 (15.4)	0.0028
Hydralazine	1297 (9.4)	1348 (9.8)	-0.0126
Loop diuretics	10781 (78.4)	10751 (78.2)	0.0053
Nitrates	2346 (17.1)	2384 (17.3)	-0.0073
Other diuretics (except loop and thiazide diuretics)	4391 (31.9)	4456 (32.4)	-0.0101
Other lipid lower therapies (excluding statins)	1332 (9.7)	1297 (9.4)	0.0087
Statins	9504 (69.1)	9492 (69.0)	0.0019
Sodium-glucose cotransporter-2 (SGLT2) inhibitors	235 (1.7)	220 (1.6)	0.0085
Thiazide diuretics	1768 (12.9)	1788 (13.0)	-0.0043
Diabetes medications			
Dipeptidyl peptidase 4 (DPP-4) inhibitors	1035 (7.5)	1040 (7.6)	-0.0014
Glucagon-like peptide 1 (GLP-1) agonists	310 (2.3)	315 (2.3)	-0.0024
Insulin	2495 (18.1)	2523 (18.3)	-0.0053
Metformin	2288 (16.6)	2332 (17.0)	-0.0086
Sulfonylureas	1700 (12.4)	1754 (12.8)	-0.0119
Other medications			
Anti-arrhythmics	2825 (20.5)	2809 (20.4)	0.0029
Anti-coagulants	6379 (46.4)	6428 (46.7)	-0.0071
Anti-platelets	3807 (27.7)	3804 (27.7)	0.0005
Non-steroidal anti-inflammatory drugs (NSAIDs)	2304 (16.8)	2332 (17.0)	-0.0054
Opioids	5382 (39.1)	5480 (39.8)	-0.0146
**Burden of comorbidities, mean (SD)**			
Combined comorbidity score	7.293 (3.267)	7.326 (3.204)	-0.0100
Frailty score	0.219 (0.056)	0.22 (0.055)	-0.0045
**Health care utilization within past 365 days**			
No. emergency department visits, mean (SD)	2.215 (2.725)	2.278 (2.641)	-0.0237
No. hospitalizations, mean (SD)			
Heart failure in primary diagnosis position	0.47 (0.881)	0.48 (0.844)	-0.0116
Heart failure NOT in primary diagnosis position	0.963 (1.391)	0.987 (1.283)	-0.0177
No. office visits, mean (SD)			
Cardiology	10.598 (10.654)	10.783 (11.712)	-0.0165
Internal medicine or family medicine	18.99 (27.788)	19.231 (26.942)	-0.0088
No. dispensed medications, mean (SD)			
Brand-name medications	4.172 (3.050)	4.179 (3.167)	-0.0023

© 2025 Hwang CS et al. *JAMA Health Forum*

Generic medications	10.132 (4.816)	10.23 (5.062)	-0.0198
Laboratory tests, imaging, and vaccination, N (%)			
Hemoglobin A1c	8066 (58.6)	8056 (58.6)	0.0015
Colon cancer screening	1849 (13.4)	1852 (13.5)	-0.0006
Creatinine	13388 (97.3)	13399 (97.4)	-0.0050
Echocardiography	12191 (88.6)	12210 (88.8)	-0.0044
Electrocardiography	12681 (92.2)	12683 (92.2)	-0.0005
Influenza vaccination	8298 (60.3)	8341 (60.6)	-0.0064
Mammography	994 (7.2)	963 (7.0)	0.0088
Microalbuminuria	2825 (20.5)	2872 (20.9)	-0.0080
Pneumococcal vaccination	2303 (16.7)	2325 (16.9)	-0.0043
Prostate cancer screening	3369 (24.5)	3421 (24.9)	-0.0088
Shingles vaccination	421 (3.1)	438 (3.2)	-0.0071
**Health care costs within past 365 days, mean (SD)**			
Log transformed total cost*	10.213 (1.052)	10.214 (1.106)	-0.0016
Total cost	44808 (48490)	46103 (52989)	-0.0255

*Log-transformed to better represent the right-skewed distribution of cost data.

© 2025 Hwang CS et al. *JAMA Health Forum*

eTable 4. Censoring reasons for our 13,775 matched pairs in our *intention-to-treat* primary analyses.

Reason for censoring	N (%)
Death	4,328 (15.73)
Disenrollment	10,746 (39.06)
End of 365-day follow-up period	12,436 (45.21)

eTable 5. Total health care and out-of-pocket costs during the 365 days following sacuitril-valsaran or ACE-I/ARB initiation using an *intention-to-treat* approach for patients *LESS THAN 80 YEARS OLD.*

	Sacubitril-Valsartan Initiators, mean annual cost (SD)*	ACE-I/ARB Initiators, mean annual cost (SD)*	Mean Cost Difference (95% CI)**	Mean Cost Ratio (95% CI)**
**Total costs <u>without</u> sacubitril-valsartan rebates*** ($)**	**38,886 (49,109)**	**38,217 (50,592)**	**-32 (-1,213 to 1,197)**	**1.00 (0.97 to 1.03)**
Inpatient costs	17,615 (38,132)	18,458 (38,671)	-1,307 (-2,218 to -291)	0.93 (0.88 to 0.98)
Outpatient costs	12,750 (19,683)	13,098 (19,004)	-576 (-1,079 to -88)	0.95 (0.92 to 0.99)
Prescription drug costs	6,890 (12,145)	5,047 (12,515)	1,864 (1,555 to 2,163)	1.39 (1.31 to 1.47)
ARNI/ACE-I/ARBs	*2,034 (1,853)*	*155 (613)*	*1,819 (1,787 to 1,849)*	*13.03 (12.00 to 14.19)*
*Other prescription drugs****	*4,856 (11,670)*	*4,892 (12,472)*	*46 (-256 to 334)*	*1.01 (0.95 to 1.08)*
**Total costs <u>with</u> sacubitril-valsartan rebates*** ($)**	**38,156 (49,094)**	**38,171 (50,851)**	**-686 (-1,868 to 547)**	**0.98 (0.95 to 1.01)**
Inpatient costs	17,615 (38,132)	18,458 (38,671)	-1,307 (-2,218 to -291)	0.93 (0.88 to 0.98)
Outpatient costs	12,750 (19,683)	13,098 (19,004)	-576 (-1,079 to -88)	0.95 (0.92 to 0.99)
Prescription drug costs	6,161 (11,943)	5,001 (12,498)	1,211 (902 to 1,506)	1.26 (1.18 to 1.33)
Sacubitril-valsartan/ACE-I/ARBs	*1,305 (1,189)*	*109 (392)*	*1,165 (1,145 to 1,185)*	*11.88 (11.03 to 12.87)*
Other prescription drugs	*4,856 (11,670)*	*4,892 (12,472)*	*46 (-256 to 334)*	*1.01 (0.95 to 1.08)*
**Out-of-pocket costs ($)**	**4,313 (5,365)**	**4,184 (5,776)**	**48 (-77 to 179)**	**1.01 (0.98 to 1.04)**
Inpatient out-of-pocket costs	1,180 (3,059)	1,166 (3,286)	11 (-71 to 99)	1.01 (0.94 to 1.09)
Outpatient out-of-pocket costs	2,293 (3,871)	2,409 (4,117)	-189 (-286 to -99)	0.92 (0.88 to 0.96)
Prescription drug out-of-pocket drug costs	841 (1,073)	608 (939)	226 (202 to 250)	1.38 (1.33 to 1.43)
ARNI/ACE-I/ARBs	*262 (380)*	*29 (110)*	*231 (225 to 237)*	*9.45 (8.75 to 10.34)*
*Other prescription drugs****	*579 (884)*	*580 (918)*	*-5 (-27 to 19)*	*0.99 (0.95 to 1.03)*

*Censoring for death or disenrollment before 365 days was accounted for based on weighting by Kaplan-Meier probabilities as described by Lin et al.[23]
**Mean differences, mean ratios, and 95% CIs were calculated using a non-parametric bootstrapping method with 500 samples drawn with replacement.
***Total costs with and without rebates include home health services, which were not included in the inpatient, outpatient, or prescription drug costs.
**** All prescription drugs <u>except</u> sacubitril-valsartan, ACE-Is, or ARBs.

© 2025 Hwang CS et al. *JAMA Health Forum*

eTable 6. Total health care and out-of-pocket costs during the 365 days following sacubitril-valsartan or ACE-I/ARB initiation using an *intention-to-treat* approach for patients *GREATER THAN OR EQUAL TO 80 YEARS OLD*.

	Sacubitril-Valsartan Initiators, mean annual cost (SD)*	ACE-I/ARB Initiators, mean annual cost (SD)*	Mean Cost Difference (95% CI)**	Mean Cost Ratio (95% CI)**
**Total costs <u>without</u> sacubitril-valsartan rebates*** ($)**	**33,773 (38,385)**	**31,739 (36,422)**	**1,858 (747 to 2,974)**	**1.06 (1.02 to 0.10)**
Inpatient costs	16,663 (29,874)	16,542 (28,149)	-57 (-941 to 877)	1.00 (0.94 to 1.05)
Outpatient costs	9,131 (13,839)	9,101 (13,614)	76 (-338 to 536)	1.01 (0.96 to 1.06)
Prescription drug costs	5,611 (11,081)	3,510 (7,925)	2,047 (1781 to 2,355)	1.31 (1.51 to 1.74)
ARNI/ACE-I/ARBs	*1,859 (1,791)*	*109 (496)*	*1,671 (1633 to 1,713)*	*17.13 (15.17 to 19.33)*
*Other prescription drugs*** **	*3,752 (10,590)*	*3,401 (7,874)*	*376 (116 to 675)*	*1.12 (1.03 to 1.22)*
**Total costs <u>with</u> sacubitril-valsartan rebates*** ($)**	**33,112 (38339)**	**31,710 (36,408)**	**1,262 (149 to 2,375)**	**1.04 (1.00 to 1.08)**
Inpatient costs	16,663 (29,874)	16,542 (28,149)	-57 (-941 to 877)	1.00 (0.94 to 1.05)
Outpatient costs	9,131 (13,839)	9,101 (13,614)	76 (-338 to 536)	1.01 (0.96 to 1.06)
Prescription drug costs	4950 (10874)	3,482 (7,905)	1,452 (1189 to 1,754)	1.44 (1.35 to 1.55)
Sacubitril-valsartan/ACE-I/ARBs	*1198 (1155)*	*81 (324)*	*1,076 (1051 to 1,103)*	15.01 *(13.45 to 16.70)*
Other prescription drugs	*3,752 (10,590)*	*3,401 (7,874)*	*376 (116 to 675)*	*1.12 (1.03 to 1.22)*
**Out-of-pocket costs ($)**	**3,774 (4,106)**	**3,551 (4,758)**	**205 (69 to 334)**	**1.06 (1.02 to 1.10)**
Inpatient out-of-pocket costs	1,270 (2,737)	1,243 (3,444)	6 (-93 to 95)	1.01 (0.93 to 1.08)
Outpatient out-of-pocket costs	1,718 (2,426)	1,746 (2,654)	-36 (-112 to 46)	0.98 (0.93 to 1.03)
Prescription drug out-of-pocket drug costs	785 (1,027)	561 (793)	234 (207 to 265)	1.44 (1.38 to 1.51)
ARNI/ACE-I/ARBs	*251 (372)*	*23 (90)*	*225 (216 to 233)*	*11.42 (10.23 to 12.78)*
*Other prescription drugs*** **	*354 (830)*	*539 (774)*	*10 (-15 to 35)*	*1.10 (0.97 to 1.07)*

*Censoring for death or disenrollment before 365 days was accounted for based on weighting by Kaplan-Meier probabilities as described by Lin et al.[23]
**Mean differences, mean ratios, and 95% CIs were calculated using a non-parametric bootstrapping method with 500 samples drawn with replacement.
***Total costs with and without rebates include home health services, which were not included in the inpatient, outpatient, or prescription drug costs.
**** All prescription drugs <u>except</u> sacubitril-valsartan, ACE-Is, or ARBs.

eTable 7. Total health care and out-of-pocket costs during the 365 days following sacubitril-valsartan or ACE-I/ARB initiation using an *intention-to-treat* approach for patients *with reported MALE SEX*.

	**Sacubitril-Valsartan Initiators, mean annual cost (SD)***	**ACE-I/ARB Initiators, mean annual cost (SD)***	**Mean Cost Difference (95% CI)****	**Mean Cost Ratio (95% CI)****
**Total costs without sacubitril-valsartan rebates*** ($)**	**36,923 (46,122)**	**35,623 (45,995)**	**204 (-1,014 to 1,290)**	**1.01 (0.97 to 1.04)**
Inpatient costs	16,944 (36,198)	17,276 (35,042)	-1,135 (-2,055 to -234)	0.94 (0.89 to 0.99)
Outpatient costs	11,968 (17,491)	12,238 (18,486)	-400 (-849 to -21)	0.97 (0.93 to 1.00)
Prescription drug costs	6,351 (11,645)	4,350 (10,999)	1,893 (1,616 to 2,174)	1.45 (1.36 to 1.54)
ARNI/ACE-I/ARBs	1,938 (1,782)	142 (587)	1,734 (1,686 to 1,771)	13.65 (12.24 to 14.99)
*Other prescription drugs*** **	4,414 (11,182)	4,208 (10,947)	135 (-189 to 454)	1.03 (0.96 to 1.12)
**Total costs with sacubitril-valsartan rebates*** ($)**	**36,231 (46,100)**	**35,581 (45,984)**	**-398 (-1,689 to 701)**	**0.99 (0.95 to 1.02)**
Inpatient costs	16,944 (36,198)	17,276 (35,042)	-1,135 (-2,055 to -234)	0.94 (0.89 to 0.99)
Outpatient costs	11,968 (17,491)	12,238 (18,486)	-400 (-849 to -21)	0.97 (0.93 to 1.00)
Prescription drug costs	5,660 (11,450)	4,308 (10,978)	1,253 (652 to 1,607)	1.30 (1.15 to 1.40)
Sacubitril-valsartan/ACE-I/ARBs	1,246 (1,147)	101 (376)	1,113 (1,052 to 1,137)	12.40 (11.12 to 13.54)
Other prescription drugs	4,414 (11,182)	4,208 (10,947)	135 (-189 to 454)	1.03 (0.96 to 1.12)
**Out-of-pocket costs ($)**	**4,142 (4,830)**	**4,027 (5,441)**	**63 (-61 to 192)**	**1.02 (0.99 to 1.05)**
Inpatient out-of-pocket costs	1,095 (2,785)	1,112 (2,973)	-40 (-115 to 36)	0.97 (0.90 to 1.03)
Outpatient out-of-pocket costs	2,155 (3,363)	2,285 (4,020)	-150 (-242 to -72)	0.93 (0.89 to 0.97)
Prescription drug out-of-pocket drug costs	893 (1,078)	630 (940)	255 (227 to 280)	1.41 (1.35 to 1.46)
ARNI/ACE-I/ARBs	281 (388)	27 (103)	249 (241 to 256)	10.35 (9.47 to 11.30)
*Other prescription drugs*** **	611 (883)	602 (922)	5 (-25 to 29)	1.01 (0.96 to 1.05)

*Censoring for death or disenrollment before 365 days was accounted for based on weighting by Kaplan-Meier probabilities as described by Lin et al.[23]

**Mean differences, mean ratios, and 95% CIs were calculated using a non-parametric bootstrapping method with 500 samples drawn with replacement.

***Total costs with and without rebates include home health services, which were not included in the inpatient, outpatient, or prescription drug costs.

**** All prescription drugs except sacubitril-valsartan, ACE-Is, or ARBs.

© 2025 Hwang CS et al. *JAMA Health Forum*

eTable 8. Total health care and out-of-pocket costs during the 365 days following sacubitril-valsartan or ACE-I/ARB initiation using an *intention-to-treat* approach for patients *with reported FEMALE SEX*.

	Sacubitril-Valsartan Initiators, mean annual cost (SD)*	ACE-I/ARB Initiators, mean annual cost (SD)*	Mean Cost Difference (95% CI)**	Mean Cost Ratio (95% CI)**
**Total costs without sacubitril-valsartan rebates*** ($)**	**36,194 (41,952)**	**35,011 (45,080)**	**1,454 (-177 to 2,848)**	**1.04 (0.99 to 1.09)**
Inpatient costs	17,585 (32,441)	17,874 (35,251)	-252 (-1,368 to 778)	0.99 (0.93 to 1.05)
Outpatient costs	9,894 (14,285)	10,528 (16,372)	-249 (-756 to 244)	0.98 (0.93 to 1.03)
Prescription drug costs	6,341 (11,593)	4,312 (9,872)	1,918 (1,570 to 2,268)	1.46 (1.36 to 1.57)
ARNI/ACE-I/ARBs	2,009 (1,906)	135 (572)	1,798 (1,756 to 1,844)	15.33 (13.52 to 17.42)
*Other prescription drugs*****	4,332 (11,073)	4,177 (9,832)	121 (-215 to 483)	1.03 (0.95 to 1.13)
**Total costs with sacubitril-valsartan rebates*** ($)**	**35,475 (41,910)**	**34,974 (45,074)**	**810 (-477 to 2,162)**	**1.02 (0.99 to 1.07)**
Inpatient costs	17,585 (32,441)	17,874 (35,251)	-252 (-1,368 to 778)	0.99 (0.93 to 1.05)
Outpatient costs	9,894 (14,285)	10,528 (16,372)	-249 (-756 to 244)	0.98 (0.93 to 1.03)
Prescription drug costs	5,621 (11,370)	4,275 (9,856)	1,275 (932 to 1,631)	1.31 (1.22 to 1.41)
Sacubitril-valsartan/ACE-I/ARBs	1,289 (1,222)	98 (370)	1,153 (1,128 to 1,181)	13.60 (12.16 to 15.23)
Other prescription drugs	4,332 (11,073)	4,177 (9,832)	121 (-215 to 483)	1.03 (0.95 to 1.13)
**Out-of-pocket costs ($)**	**3,950 (4,484)**	**3,874 (5,756)**	**172 (10 to 339)**	**1.05 (1.00 to 1.09)**
Inpatient out-of-pocket costs	1,408 (3,157)	1,350 (4,070)	88 (-33 to 201)	1.07 0.98 to 1.16)
Outpatient out-of-pocket costs	1,846 (2,527)	2,019 (3,428)	-99 (-195 to -2)	0.95 (0.90 to 1.00)
Prescription drug out-of-pocket drug costs	696 (1,002)	506 (798)	182 (153 to 209)	1.37 (1.29 to 1.43)
ARNI/ACE-I/ARBs	219 (356)	24 (98)	193 (185 to	9.75 (8.75 to 11.07)
*Other prescription drugs*****	477 (811)	482 (779)	-11 (-37 to 12)	0.98 (0.93 to 1.03)

*Censoring for death or disenrollment before 365 days was accounted for based on weighting by Kaplan-Meier probabilities as described by Lin et al.[23]

**Mean differences, mean ratios, and 95% CIs were calculated using a non-parametric bootstrapping method with 500 samples drawn with replacement.

***Total costs with and without rebates include home health services, which were not included in the inpatient, outpatient, or prescription drug costs.

**** All prescription drugs except sacubitril-valsartan, ACE-Is, or ARBs.

© 2025 Hwang CS et al. *JAMA Health Forum*

eTable 9. Total health care and out-of-pocket costs during the 365 days following sacubitril-valsartan or ACE-I/ARB initiation using an *intention-to-treat* approach for patients *who reported WHITE RACE*.

	**Sacubitril-Valsartan Initiators, mean annual cost (SD)***	**ACE-I/ARB Initiators, mean annual cost (SD)***	**Mean Cost Difference (95% CI)****	**Mean Cost Ratio (95% CI)****
**Total costs without sacubitril-valsartan rebates*** ($)**	**35,809 (43,894)**	**35,215 (45,848)**	**574 (-333 to 1,520)**	**1.02 (0.99 to 1.04)**
Inpatient costs	16,405 (34,004)	17,472 (35,714)	-941 (-1,689 to -113)	0.95 (0.90 to 0.99)
Outpatient costs	11,189 (16,982)	11,487 (17,314)	-289 (-617 to 32)	0.97 (0.94 to 1.00)
Prescription drug costs	6,344 (11,638)	4,297 (10,924)	1,899 (1,665 to 2,129)	1.46 (1.39 to 1.54)
ARNI/ACE-I/ARBs	1,969 (1,813)	133 (566)	1,757 (1,729 to 1,783)	14.45 (13.37 to 15.67)
*Other prescription drugs****	4,375 (11,158)	4,163 (10,885)	142 (-95 to 365)	1.04 (0.98 to 1.10)
**Total costs with sacubitril-valsartan rebates*** ($)**	**35,106 (43,868)**	**35,178 (45,840)**	**-55 (-965 to 891)**	**1.00 (0.97 to 1.03)**
Inpatient costs	16,405 (34,004)	17,472 (35,714)	-941 (-1,689 to -113)	0.95 (0.90 to 0.99)
Outpatient costs	11,189 (16,982)	11,487 (17,314)	-289 (-617 to 32)	0.97 (0.94 to 1.00)
Prescription drug costs	5,641 (11,436)	4,259 (10,908)	1,271 (1,035 to 1,494)	1.31 (1.24 to 1.38)
Sacubitril-valsartan/ACE-I/ARBs	1,266 (1,167)	96 (366)	1,128 (1,111 to 1,145)	13.00 (12.14 to 13.97)
Other prescription drugs	4,375 (11,158)	4,163 (10,885)	142 (-95 to 365)	1.04 (0.98 to 1.10)
**Out-of-pocket costs ($)**	**4,081 (4,707)**	**4,000 (5,500)**	**103 (-3 to 216)**	**1.03 (1.00 to 1.06)**
Inpatient out-of-pocket costs	1,149 (2,717)	1,209 (3,467)	-23 (-94 to 51)	0.98 (0.92 to 1.04)
Outpatient out-of-pocket costs	2,031 (3,268)	2,147 (3,682)	-125 (-193 to -56)	0.94 (0.91 to 0.97)
Prescription drug out-of-pocket drug costs	901 (1,079)	644 (941)	251 (231 to 272)	1.40 (1.36 to 1.44)
ARNI/ACE-I/ARBs	285 (391)	27 (102)	251 (246 to 257)	10.37 (9.72 to 11.15)
*Other prescription drugs****	616 (884)	617 (924)	0 (-19 to 18)	1.00 (0.97 to 1.03)

*Censoring for death or disenrollment before 365 days was accounted for based on weighting by Kaplan-Meier probabilities as described by Lin et al.[23]
**Mean differences, mean ratios, and 95% CIs were calculated using a non-parametric bootstrapping method with 500 samples drawn with replacement.
***Total costs with and without rebates include home health services, which were not included in the inpatient, outpatient, or prescription drug costs.
**** All prescription drugs except sacubitril-valsartan, ACE-Is, or ARBs.

© 2025 Hwang CS et al. *JAMA Health Forum*

eTable 10. Total health care and out-of-pocket costs during the 365 days following sacubitril-valsartan or ACE-I/ARB initiation using an *intention-to-treat* approach for patients *who reported ASIAN, BLACK, HISPANIC, or OTHER RACE*.

	Sacubitril-Valsartan Initiators, mean annual cost (SD)*	ACE-I/ARB Initiators, mean annual cost (SD)*	Mean Cost Difference (95% CI)**	Mean Cost Ratio (95% CI)**
**Total costs without sacubitril-valsartan rebates*** ($)**	**41,360 (49,768)**	**39,581 (51,172)**	**833 (-1,609 to 3,304)**	**1.02 (0.96 to 1.09)**
Inpatient costs	20,217 (37,456)	20,309 (38,748)	-387 (-2,238 to 1,659)	0.98 (0.90 to 1.08)
Outpatient costs	1,300 (3,174)	12,331 (19,662)	-570 (-1,492 to 332)	0.95 (0.88 to 1.03)
Prescription drug costs	6,847 (13,154)	4,797 (11,200)	1,901 (1,251 to 2,545)	1.41 (1.25 to 1.58)
ARNI/ACE-I/ARBs	2,000 (1,930)	149 (624)	1,783 (1,709 to 1,852)	13.45 (11.28 to 15.99)
*Other prescription drugs****	4,847 (12,632)	4,648 (11,147)	119 (-510 to 736)	1.03 (0.90 to 1.17)
**Total costs with sacubitril-valsartan rebates*** ($)**	**40,638 (49,728)**	**39,537 (51,162)**	**191 (-2,257 to 2,656)**	**1.01 (0.95 to 1.07)**
Inpatient costs	20,217 (37,456)	20,309 (38,748)	-387 (-2,238 to 1,659)	0.98 (0.90 to 1.08)
Outpatient costs	1,300 (3,174)	12,331 (19,662)	-570 (-1,492 to 332)	0.95 (0.88 to 1.03)
Prescription drug costs	6,126 (12,932)	4,754 (11,178)	1,260 (620 to 1,885)	1.27 (1.12 to 1.27)
Sacubitril-valsartan/ACE-I/ARBs	1,279 (1,231)	106 (401)	1,141 (1,095 to 1,186)	12.24 (10.41 to 14.30)
Other prescription drugs	4,847 (12,632)	4,648 (11,147)	119 (-510 to 736)	1.03 (0.90 to 1.17)
**Out-of-pocket costs ($)**	**4,132 (5,770)**	**3,935 (5,174)**	**112 (-161 to 400)**	**1.03 (0.96 to 1.11)**
Inpatient out-of-pocket costs	1,445 (3,662)	1,300 (3,174)	156 (-37 to 335)	1.12 (0.97 to 1.28)
Outpatient out-of-pocket costs	2,249 (4,031)	2,325 (3,524)	-155 (-329 to -1)	0.93 (0.86 to 1.00)
Prescription drug out-of-pocket drug costs	438 (904)	310 (630)	111 (75 to 143)	1.35 (1.23 to 1.48)
ARNI/ACE-I/ARBs	129 (266)	16 (66)	110 (101 to 119)	7.68 (6.47 to 9.04)
*Other prescription drugs****	309 (774)	294 (613)	1 (-32 to 30)	1.00 (0.90 to 1.10)

*Censoring for death or disenrollment before 365 days was accounted for based on weighting by Kaplan-Meier probabilities as described by Lin et al.[23]
**Mean differences, mean ratios, and 95% CIs were calculated using a non-parametric bootstrapping method with 500 samples drawn with replacement.
***Total costs with and without rebates include home health services, which were not included in the inpatient, outpatient, or prescription drug costs.
**** All prescription drugs except sacubitril-valsartan, ACE-Is, or ARBs.

© 2025 Hwang CS et al. *JAMA Health Forum*

eTable 11. Total health care and out-of-pocket costs during the 365 days following sacubitril-valsartan or ACE-I/ARB initiation using an *intention-to-treat* approach for patients *with LOW FRAILTY SCORES (frailty score < 0.15)*.

	Sacubitril-Valsartan Initiators, mean annual cost (SD)*	ACE-I/ARB Initiators, mean annual cost (SD)*	Mean Cost Difference (95% CI)**	Mean Cost Ratio (95% CI)**
**Total costs without sacubitril-valsartan rebates*** ($)**	**23,417 (38,102)**	**22,743 (34,348)**	**-121 (-3,400 to 2,910)**	**1.00 (0.86 to 1.14)**
Inpatient costs	6,749 (20,941)	7,780 (20,478)	-1,313 (-3,587 to 711)	0.84 (0.62 to 1.12)
Outpatient costs	10,974 (24,284)	11,320 (19,399)	-366 (-1,848 to 1,138)	0.97 (0.83 to 1.12)
Prescription drug costs	5,295 (14,397)	3,161 (11,516)	1,647 (718 to 2,620)	1.52 (1.19 to 1.94)
ARNI/ACE-I/ARBs	2,235 (1,793)	190 (706)	2,013 (1,904 to 2,122)	12.80 (10.10 to 16.67)
*Other prescription drugs*** **	3,060 (14,108)	2,971 (11,486)	-366 (-1,267 to 577)	0.89 (0.65 to 1.23)
**Total costs with sacubitril-valsartan rebates*** ($)**	**22,613 (38,057)**	**22,685 (34,333)**	**-849 (-4,162 to 2,149)**	**0.96 (0.83 to 1.11)**
Inpatient costs	6,749 (20,941)	7,780 (20,478)	-1,313 (-3,587 to 711)	0.84 (0.62 to 1.12)
Outpatient costs	10,974 (24,284)	11,320 (19,399)	-366 (-1,848 to 1,138)	0.97 (0.83 to 1.12)
Prescription drug costs	4,492 (14,265)	3,104 (11,501)	919 (8 to 1,880)	1.30 (1.00 to 1.68)
Sacubitril-valsartan/ACE-I/ARBs	1,431 (1,148)	133 (451)	1,286 (1,216 to 1,353)	11.69 (9.43 to 14.90)
Other prescription drugs	3,060 (14,108)	2,971 (11,486)	-366 (-1,267 to 577)	0.89 (0.65 to 1.23)
**Out-of-pocket costs ($)**	**3,178 (5,244)**	**3,013 (3,803)**	**76 (-265 to 375)**	**1.03 (0.92 to 1.15)**
Inpatient out-of-pocket costs	406 (974)	489 (1,343)	-106 (-218 to 3)	0.79 (0.60 to 1.01)
Outpatient out-of-pocket costs	1,885 (4,777)	1,945 (3,096)	-97 (-304 to 148)	0.95 (0.83 to 1.09)
Prescription drug out-of-pocket drug costs	887 (1,136)	579 (924)	279 (191 to 365)	1.48 (1.30 to 1.68)
ARNI/ACE-I/ARBs	373 (426)	42 (152)	328 (304 to 353)	9.76 (7.67 to 12.48)
*Other prescription drugs*** **	515 (965)	537 (898)	-49 (-134 to 27)	0.91 (0.78 to 1.05)

*Censoring for death or disenrollment before 365 days was accounted for based on weighting by Kaplan-Meier probabilities as described by Lin et al.[23]

**Mean differences, mean ratios, and 95% CIs were calculated using a non-parametric bootstrapping method with 500 samples drawn with replacement.

***Total costs with and without rebates include home health services, which were not included in the inpatient, outpatient, or prescription drug costs.

**** All prescription drugs except sacubitril-valsartan, ACE-Is, or ARBs.

© 2025 Hwang CS et al. *JAMA Health Forum*

eTable 12. Total health care and out-of-pocket costs during the 365 days following sacubitril-valsartan or ACE-I/ARB initiation using an *intention-to-treat* approach for patients *with MODERATE FRAILTY SCORES (0.15 ≤ frailty score < 0.25)*.

	Sacubitril-Valsartan Initiators, mean annual cost (SD)*	ACE-I/ARB Initiators, mean annual cost (SD)*	Mean Cost Difference (95% CI)**	Mean Cost Ratio (95% CI)**
**Total costs <u>without</u> sacubitril-valsartan rebates*** ($)**	**33,883 (42,924)**	**33,161 (45,193)**	**839 (-242 to 1,914)**	**1.03 (0.99 to 1.06)**
Inpatient costs	14,983 (33,521)	15,614 (34,115)	-584 (-1,459 to 291)	0.96 (0.91 to 1.02)
Outpatient costs	11,226 (16,453)	11,736 (18,605)	-422 (-808 to -70)	0.96 (0.93 to 0.99)
Prescription drug costs	6,215 (11,423)	4,263 (11,222)	1,930 (1,685 to 2,183)	1.47 (1.40 to 1.56)
ARNI/ACE-I/ARBs	2,050 (1,828)	152 (615)	1,846 (1,814 to 1,879)	13.60 (12.56 to 14.72)
*Other prescription drugs**** *	4,165 (10,960)	4,112 (11,170)	84 (-156 to 335)	1.02 (0.96 to 1.09)
**Total costs <u>with</u> sacubitril-valsartan rebates*** ($)**	**33,152 (42,901)**	**33,117 (45,183)**	**178 (-903 to 1,258)**	**1.01 (0.97 to 1.04)**
Inpatient costs	14,983 (33,521)	15,614 (34,115)	-584 (-1,459 to 291)	0.96 (0.91 to 1.02)
Outpatient costs	11,226 (16,453)	11,736 (18,605)	-422 (-808 to -70)	0.96 (0.93 to 0.99)
Prescription drug costs	5,484 (11,225)	4,219 (11,201)	1,268 (1,027 to 1,514)	1.31 (1.24 to 1.39)
Sacubitril-valsartan/ACE-I/ARBs	1,318 (1,176)	108 (394)	1,185 (1,163 to 1,207)	12.32 (11.42 to 13.22)
Other prescription drugs	4,165 (10,960)	4,112 (11,170)	84 (-156 to 335)	1.02 (0.96 to 1.09)
**Out-of-pocket costs ($)**	**3,871 (4,526)**	**3,719 (5,223)**	**134 (37 to 244)**	**1.04 (1.01 to 1.07)**
Inpatient out-of-pocket costs	978 (2,565)	926 (2,574)	41 (-24 to 108)	1.05 (0.98 to 1.12)
Outpatient out-of-pocket costs	2,023 (3,159)	2,171 (4,063)	-150 (-229 to -86)	0.93 (0.89 to 0.96)
Prescription drug out-of-pocket drug costs	870 (1,049)	622 (922)	243 (222 to 265)	1.40 (1.36 to 1.44)
ARNI/ACE-I/ARBs	284 (389)	29 (106)	250 (244 to 257)	9.95 (9.28 to 10.69)
*Other prescription drugs**** *	586 (853)	593 (904)	-7 (-25 to 13)	0.99 (0.96 to 1.02)

*Censoring for death or disenrollment before 365 days was accounted for based on weighting by Kaplan-Meier probabilities as described by Lin et al.[23]
**Mean differences, mean ratios, and 95% CIs were calculated using a non-parametric bootstrapping method with 500 samples drawn with replacement.
***Total costs with and without rebates include home health services, which were not included in the inpatient, outpatient, or prescription drug costs.
**** All prescription drugs <u>except</u> sacubitril-valsartan, ACE-Is, or ARBs.

© 2025 Hwang CS et al. *JAMA Health Forum*

eTable 13. Total health care and out-of-pocket costs during the 365 days following sacubitril-valsartan or ACE-I/ARB initiation using an *intention-to-treat* approach for patients *with HIGH FRAILTY SCORES (frailty score ≥ 0.25)*.

	**Sacubitril-Valsartan Initiators, mean annual cost (SD)***	**ACE-I/ARB Initiators, mean annual cost (SD)***	**Mean Cost Difference (95% CI)****	**Mean Cost Ratio (95% CI)****
**Total costs without sacubitril-valsartan rebates*** ($)**	**46,491 (50,455)**	**45,352 (50,420)**	**1,022 (-675 to 2,901)**	**1.02 (0.99 to 1.07)**
Inpatient costs	24,521 (39,933)	24,945 (39,615)	-591 (-2,088 to 912)	0.98 (0.92 to 1.04)
Outpatient costs	11,727 (18,911)	11,935 (17,159)	-146 (-793 to 503)	0.99 (0.93 to 1.05)
Prescription drug costs	6,981 (11,384)	5,124 (11,495)	1,810 (1,395 to 2,221)	1.39 (1.28 to 1.50)
ARNI/ACE-I/ARBs	1,742 (1,817)	104 (475)	1,514 (1,472 to 1,554)	16.57 (14.45 to 19.24)
*Other prescription drugs**** *	5,239 (10,761)	5,019 (11,447)	296 (-120 to 702)	1.07 (0.98 to 1.16)
**Total costs with sacubitril-valsartan rebates*** ($)**	**45,866 (50,402)**	**45,324 (50,410)**	**482 (-1,207 to 2,358)**	**1.01 (0.97 to 1.06)**
Inpatient costs	24,521 (39,933)	24,945 (39,615)	-591 (-2,088 to 912)	0.98 (0.92 to 1.04)
Outpatient costs	11,727 (18,911)	11,935 (17,159)	-146 (-793 to 503)	0.99 (0.93 to 1.05)
Prescription drug costs	6,355 (11,131)	5,096 (11,477)	1,270 (853 to 1,687)	1.27 (1.17 to 1.38)
Sacubitril-valsartan/ACE-I/ARBs	1,116 (1,165)	76 (307)	974 (946 to 1,000)	14.65 (12.91 to 16.67)
Other prescription drugs	5,239 (10,761)	5,019 (11,447)	296 (-120 to 702)	1.07 (0.98 to 1.16)
**Out-of-pocket costs ($)**	**4,846 (5,646)**	**4,781 (5,694)**	**48 (-181 to 261)**	**1.01 (0.96 to 1.06)**
Inpatient out-of-pocket costs	1,916 (3,782)	1,910 (4,046)	-33 (-204 to 147)	0.98 (0.90 to 1.08)
Outpatient out-of-pocket costs	2,223 (3,601)	2,349 (3,276)	-93 (-210 to 24)	0.96 (0.91 to 1.01)
Prescription drug out-of-pocket drug costs	707 (1,046)	522 (841)	174 (139 to 204)	1.34 (1.26 to 1.41)
ARNI/ACE-I/ARBs	181 (324)	17 (76)	159 (151 to 167)	10.46 (8.93 to 12.19)
*Other prescription drugs**** *	526 (862)	504 (824)	15 (-16 to 43)	1.03 (0.97 to 1.09)

*Censoring for death or disenrollment before 365 days was accounted for based on weighting by Kaplan-Meier probabilities as described by Lin et al.[23]
**Mean differences, mean ratios, and 95% CIs were calculated using a non-parametric bootstrapping method with 500 samples drawn with replacement.
***Total costs with and without rebates include home health services, which were not included in the inpatient, outpatient, or prescription drug costs.
**** All prescription drugs except sacubitril-valsartan, ACE-Is, or ARBs.

© 2025 Hwang CS et al. *JAMA Health Forum*

eTable 14. Total health care and out-of-pocket costs during the 365 days following sacubitril-valsartan or ACE-I/ARB initiation using an *intention-to-treat* approach for patients *who are LOW-INCOME SUBSIDY RECIPIENTS*.

	Sacubitril-Valsartan Initiators, mean annual cost (SD)*	ACE-I/ARB Initiators, mean annual cost (SD)*	Mean Cost Difference (95% CI)**	Mean Cost Ratio (95% CI)**
**Total costs without sacubitril-valsartan rebates*** ($)**	**43,112 (46,935)**	**41,188 (50,254)**	**1,102 (-893 to 3,026)**	**1.03 (0.98 to 1.08)**
Inpatient costs	21,230 (36,700)	21,680 (39,687)	-1,017 (-2,694 to 478)	0.96 (0.88 to 1.02)
Outpatient costs	10,845 (16,670)	10,960 (15,843)	-203 (-848 to 471)	0.98 (0.92 to 1.05)
Prescription drug costs	8,724 (12,015)	6,238 (12,447)	2,386 (1,963 to 2,835)	1.42 (1.32 to 1.52)
ARNI/ACE-I/ARBs	2,377 (2,167)	145 (652)	2,067 (2,008 to 2,126)	16.35 (14.19 to 19.01)
*Other prescription drugs**** *	6,347 (11,279)	6,093 (12,380)	319 (-80 to 757)	1.06 (0.99 to 1.14)
**Total costs with sacubitril-valsartan rebates*** ($)**	**42,259 (46,896)**	**41,146 (50,246)**	**368 (-1,626 to 2,276)**	**1.01 (0.96 to 1.06)**
Inpatient costs	21,230 (36,700)	21,680 (39,687)	-1,017 (-2,694 to 478)	0.96 (0.88 to 1.02)
Outpatient costs	10,845 (16,670)	10,960 (15,843)	-203 (-848 to 471)	0.98 (0.92 to 1.05)
Prescription drug costs	7,871 (11,709)	6,195 (12,421)	1,652 (1,239 to 2,098)	1.29 (1.21 to 1.39)
Sacubitril-valsartan/ACE-I/ARBs	1,524 (1,387)	102 (416)	1,333 (1,293 to 1,372)	14.94 (13.14 to 17.17)
Other prescription drugs	6,347 (11,279)	6,093 (12,380)	319 (-80 to 757)	1.06 (0.99 to 1.14)
**Out-of-pocket costs ($)**	**3,839 (5,004)**	**3,760 (5,171)**	**11 (-200 to 223)**	**1.00 (0.95 to 1.06)**
Inpatient out-of-pocket costs	1,680 (3,590)	1,559 (3,754)	79 (-85 to 229)	1.05 (0.95 to 1.15)
Outpatient out-of-pocket costs	2,067 (3,028)	2,113 (2,974)	-75 (-204 to 41)	0.96 (0.90 to 1.02)
Prescription drug out-of-pocket drug costs	92 (212)	89 (270)	6 (-3 to 15)	1.08 (0.96 to 1.20)
ARNI/ACE-I/ARBs	22 (70)	4 (13)	17 (16 to 19)	5.46 (4.70 to 6.21)
*Other prescription drugs**** *	70 (173)	85 (265)	-11 (-20 to -3)	0.86 (0.77 to 0.96)

*Censoring for death or disenrollment before 365 days was accounted for based on weighting by Kaplan-Meier probabilities as described by Lin et al.[23]
**Mean differences, mean ratios, and 95% CIs were calculated using a non-parametric bootstrapping method with 500 samples drawn with replacement.
***Total costs with and without rebates include home health services, which were not included in the inpatient, outpatient, or prescription drug costs.
**** All prescription drugs except sacubitril-valsartan, ACE-Is, or ARBs.

© 2025 Hwang CS et al. *JAMA Health Forum*

eTable 15. Total health care and out-of-pocket costs during the 365 days following sacubitril-valsartan or ACE-I/ARB initiation using an *intention-to-treat* approach for patients *who are NOT LOW-INCOME SUBSIDY RECIPIENTS.*

	Sacubitril-Valsartan Initiators, mean annual cost (SD)*	ACE-I/ARB Initiators, mean annual cost (SD)*	Mean Cost Difference (95% CI)**	Mean Cost Ratio (95% CI)**
**Total costs without sacubitril-valsartan rebates*** ($)**	**33,793 (44,117)**	**33,929 (44,753)**	**466 (-579 to 1,412)**	**1.01 (0.98 to 1.04)**
Inpatient costs	15,582 (33,794)	16,285 (34,063)	-704 (-1,574 to 79)	0.96 (0.91 to 1.01)
Outpatient costs	11,553 (18,224)	12,038 (18,476)	-453 (-836 to -22)	0.96 (0.93 to 1.00)
Prescription drug costs	5,528 (11,548)	3,738 (10,534)	1,731 (1,484 to 1,959)	1.48 (1.39 to 1.56)
ARNI/ACE-I/ARBs	1,816 (1,663)	134 (543)	1,644 (1,616 to 1,669)	13.43 (12.45 to 14.45)
*Other prescription drugs*** **	3,712 (11,192)	3,604 (10,488)	87 (-155 to 307)	1.03 (0.96 to 1.09)
**Total costs with sacubitril-valsartan rebates*** ($)**	**33,793 (44,117)**	**33,891 (44,741)**	**-125 (-1,167 to 818)**	**1.00 (0.97 to 1.03)**
Inpatient costs	15,582 (33,794)	16,285 (34,063)	-704 (-1,574 to 79)	0.96 (0.91 to 1.01)
Outpatient costs	11,553 (18,224)	12,038 (18,476)	-453 (-836 to -22)	0.96 (0.93 to 1.00)
Prescription drug costs	4,880 (11,393)	3,701 (10,515)	1,140 (898 to 1,361)	1.32 (1.24 to 1.40)
Sacubitril-valsartan/ACE-I/ARBs	1,168 (1,072)	97 (349)	1,053 (1,035 to 1,069)	12.05 (11.26 to 12.87)
Other prescription drugs	3,712 (11,192)	3,604 (10,488)	87 (-155 to 307)	1.03 (0.96 to 1.09)
**Out-of-pocket costs ($)**	**4,202 (4,854)**	**4,074 (5,524)**	**138 (32 to 248)**	**1.04 (1.01 to 1.06)**
Inpatient out-of-pocket costs	1,030 (2,557)	1,070 (3,216)	-704 (-1,574 to 79)	0.99 (0.93 to 1.05)
Outpatient out-of-pocket costs	2,070 (3,541)	2,218 (3,899)	-453 (-836 to -22)	0.92 (0.89 to 0.96)
Prescription drug out-of-pocket drug costs	1,102 (1,114)	786 (982)	312 (289 to 335)	1.41 (1.37 to 1.44)
ARNI/ACE-I/ARBs	348 (407)	34 (117)	1,644 (1,616 to 1,669)	10.24 (9.59 to 10.92)
*Other prescription drugs*** **	754 (942)	752 (965)	4 (-17 to 24)	1.01 (0.98 to 1.03)

*Censoring for death or disenrollment before 365 days was accounted for based on weighting by Kaplan-Meier probabilities as described by Lin et al.[23]
**Mean differences, mean ratios, and 95% CIs were calculated using a non-parametric bootstrapping method with 500 samples drawn with replacement.
***Total costs with and without rebates include home health services, which were not included in the inpatient, outpatient, or prescription drug costs.
**** All prescription drugs except sacubitril-valsartan, ACE-Is, or ARBs.

© 2025 Hwang CS et al. *JAMA Health Forum*

eTable 16. Total health care and out-of-pocket costs during the 365 days following sacubitril-valsartan or ACE-I/ARB initiation using an *intention-to-treat* approach for patients *with AT LEAST ONE HEART FAILURE HOSPITALIZATION during the baseline period*.

	**Sacubitril-Valsartan Initiators, mean annual cost (SD)***	**ACE-I/ARB Initiators, mean annual cost (SD)***	**Mean Cost Difference (95% CI)****	**Mean Cost Ratio (95% CI)****
**Total costs <u>without</u> sacubitril-valsartan rebates*** ($)**	**44,263 (48,824)**	**44,468 (50,201)**	**-1,018 (-2,769 to 857)**	**0.98 (0.94 to 1.02)**
Inpatient costs	23,669 (39,596)	24,917 (40,515)	-1,724 (-3,144 to -233)	0.93 (0.88 to 0.99)
Outpatient costs	11,716 (16,700)	12,297 (16,855)	-835 (-1,456 to -196)	0.93 (0.88 to 0.98)
Prescription drug costs	6,089 (10,119)	4,350 (10,506)	1,623 (1,275 to 1,996)	1.39 (1.28 to 1.50)
ARNI/ACE-I/ARBs	*1,780 (1,799)*	*138 (603)*	*1,565 (1,522 to 1,606)*	*12.45 (10.77 to 14.19)*
*Other prescription drugs*****	*4,309 (9,534)*	*4,212 (10,452)*	*58 (-300 to 414)*	*1.02 (0.93 to 1.11)*
**Total costs <u>with</u> sacubitril-valsartan rebates*** ($)**	**43,625 (48,794)**	**44,426 (50,192)**	**-1,578 (-3,323 to 289)**	**0.96 (0.93 to 1.01)**
Inpatient costs	23,669 (39,596)	24,917 (40,515)	-1,724 (-3,144 to -233)	0.93 (0.88 to 0.99)
Outpatient costs	11,716 (16,700)	12,297 (16,855)	-835 (-1,456 to -196)	0.93 (0.88 to 0.98)
Prescription drug costs	*5,450 (9,874)*	*4,309 (10,484)*	*1,063 (714 to 1,429)*	*1.26 (1.16 to 1.36)*
Sacubitril-valsartan/ACE-I/ARBs	*1,141 (1,152)*	*97 (386)*	*1,005 (978 to 1,031)*	*11.47 (10.05 to 12.95)*
Other prescription drugs	*4,309 (9,534)*	*4,212 (10,452)*	*58 (-300 to 414)*	*1.02 (0.93 to 1.11)*
**Out-of-pocket costs ($)**	**4,515 (4,969)**	**4,507 (5,361)**	**-87 (-277 to 114)**	**0.98 (0.94 to 1.03)**
Inpatient out-of-pocket costs	1,625 (3,439)	1,622 (3,789)	-37 (-187 to 93)	0.98 (0.90 to 1.06)
Outpatient out-of-pocket costs	2,143 (2,914)	2,333 (3,111)	-243 (-368 to -129)	0.89 (0.84 to 0.94)
Prescription drug out-of-pocket drug costs	748 (1010)	553 (854)	193 (163 to 225)	1.36 (1.29 to 1.44)
ARNI/ACE-I/ARBs	*218 (350)*	*23 (96)*	*189 (182 to 197)*	*8.96 (7.90 to 10.24)*
*Other prescription drugs*****	*530 (815)*	*530 (836)*	*4 (-26 to 33)*	*0.01 (0.95 to 1.07)*

*Censoring for death or disenrollment before 365 days was accounted for based on weighting by Kaplan-Meier probabilities as described by Lin et al.[23]

**Mean differences, mean ratios, and 95% CIs were calculated using a non-parametric bootstrapping method with 500 samples drawn with replacement.

***Total costs with and without rebates include home health services, which were not included in the inpatient, outpatient, or prescription drug costs.

**** All prescription drugs <u>except</u> sacubitril-valsartan, ACE-Is, or ARBs.

© 2025 Hwang CS et al. *JAMA Health Forum*

eTable 17. Total health care and out-of-pocket costs during the 365 days following sacubitril-valsartan or ACE-I/ARB initiation using an *intention-to-treat* approach for patients *with NO HEART FAILURE HOSPITALIZATION during the baseline period*.

	Sacubitril-Valsartan Initiators, mean annual cost (SD)*	ACE-I/ARB Initiators, mean annual cost (SD)*	Mean Cost Difference (95% CI)**	Mean Cost Ratio (95% CI)**
**Total costs without sacubitril-valsartan rebates*** ($)**	**33,295 (42,830)**	**31,893 (43,774)**	**1,489 (498 to 2,590)**	**1.05 (1.02 to 1.09)**
Inpatient costs	14,182 (32,176)	14,444 (32,291)	-242 (-1,043 to 617)	0.98 (0.93 to 1.05)
Outpatient costs	11,094 (18,054)	11,290 (17,805)	-160 (-580 to 275)	0.99 (0.95 to 1.03)
Prescription drug costs	6,503 (12,412)	4,573 (12,190)	1,958 (1,683 to 2,243)	1.46 (1.38 to 1.53)
ARNI/ACE-I/ARBs	*2,052 (1,839)*	*139 (578)*	*1,850 (1,821 to 1,879)*	*15.28 (14.04 to 16.52)*
*Other prescription drugs**** *	*4,451 (11,974)*	*4,435 (12,141)*	*108 (-163 to 379)*	*1.03 (0.96 to 1.09)*
**Total costs with sacubitril-valsartan rebates*** ($)**	**32,562 (42,801)**	**31,855 (43,762)**	**826 (-162 to 1,927)**	**1.03 (0.99 to 1.06)**
Inpatient costs	14,182 (32,176)	14,444 (32,291)	-242 (-1,043 to 617)	0.98 (0.93 to 1.05)
Outpatient costs	11,094 (18,054)	11,290 (17,805)	-160 (-580 to 275)	0.99 (0.95 to 1.03)
Prescription drug costs	5,770 (12,225)	4,535 (12,171)	1,295 (1,025 to 1,576)	1.31 (1.23 to 1.38)
Sacubitril-valsartan/ACE-I/ARBs	*1,319 (1,183)*	*100 (372)*	*1,188 (1,168 to 1,206)*	*13.60 (12.63 to 14.58)*
Other prescription drugs	*4,451 (11,974)*	*4,435 (12,141)*	*108 (-163 to 379)*	*1.03 (0.96 to 1.09)*
**Out-of-pocket costs ($)**	**3,906 (4897)**	**3,730 (5,157)**	**175 (65 to 287)**	**1.05 (1.02 to 1.08)**
Inpatient out-of-pocket costs	1,033 (2689)	1,014 (2,781)	28 (-38 to 97)	1.03 (0.96 to 1.10)
Outpatient out-of-pocket costs	2,018 (3594)	2,096 (3,802)	-94 (-170 to -14)	0.95 (0.92 to 0.99)
Prescription drug out-of-pocket drug costs	855 (1078)	621 (948)	241 (217 to 262)	1.40 (1.35 to 1.45)
ARNI/ACE-I/ARBs	*277 (387)*	*27 (98)*	*247 (241 to 253)*	*10.67 (9.94 to 11.46)*
*Other prescription drugs**** *	*578 (889)*	*594 (931)*	*-6 (-27 to 14)*	*0.99 (0.95 to 1.03)*

*Censoring for death or disenrollment before 365 days was accounted for based on weighting by Kaplan-Meier probabilities as described by Lin et al.[23]

**Mean differences, mean ratios, and 95% CIs were calculated using a non-parametric bootstrapping method with 500 samples drawn with replacement.

***Total costs with and without rebates include home health services, which were not included in the inpatient, outpatient, or prescription drug costs.

**** All prescription drugs except sacubitril-valsartan, ACE-Is, or ARBs.

© 2025 Hwang CS et al. *JAMA Health Forum*

eTable 18. Total health care and out-of-pocket costs during the 365 days following sacubitril-valsartan or ACE-I/ARB initiation using an *intention-to-treat* approach for patients *with baseline health care costs in the LOWEST quartile*.

	Sacubitril-Valsartan Initiators, mean annual cost (SD)*	ACE-I/ARB Initiators, mean annual cost (SD)*	Mean Cost Difference (95% CI)**	Mean Cost Ratio (95% CI)**
**Total costs without sacubitril-valsartan rebates*** ($)**	**22,878 (31,071)**	**20,415 (28,713)**	**2,560 (1,396 to 3,705)**	**1.13 (1.07 to 1.19)**
Inpatient costs	9,952 (25,576)	9,132 (22,501)	834 (-200 to 1,801)	1.09 (0.98 to 1.21)
Outpatient costs	7,825 (11,679)	8,005 (12,494)	-110 (-515 to 327)	0.99 (0.93 to 1.05)
Prescription drug costs	4,191 (4,711)	2,379 (4,986)	1,807 (1,620 to 1,980)	1.77 (1.66 to 1.89)
ARNI/ACE-I/ARBs	2,091 (1,795)	130 (534)	1,923 (1,870 to 1,972)	16.54 (14.61 to 19.00)
*Other prescription drugs**** *	2,100 (4,063)	2,249 (4,937)	-116 (-285 to 56)	0.95 (0.88 to 1.03)
**Total costs with sacubitril-valsartan rebates*** ($)**	**22,137 (31,041)**	**20,380 (28,697)**	**1,875 (717 to 3,012)**	**1.09 (1.03 to 1.16)**
Inpatient costs	9,952 (25,576)	9,132 (22,501)	834 (-200 to 1,801)	1.09 (0.98 to 1.21)
Outpatient costs	7,825 (11,679)	8,005 (12,494)	-110 (-515 to 327)	0.99 (0.93 to 1.05)
Prescription drug costs	3,449 (4,412)	2,344 (4,963)	1,122 (942 to 1,289)	1.49 (1.39 to 1.59)
Sacubitril-valsartan/ACE-I/ARBs	1,349 (1,162)	96 (348)	1,238 (1,204 to 1,270)	14.52 (13.01 to 16.44)
Other prescription drugs	2,100 (4,063)	2,249 (4,937)	-116 (-285 to 56)	0.95 (0.88 to 1.03)
**Out-of-pocket costs ($)**	**2,861 (3,217)**	**2,562 (3,201)**	**272 (158 to 390)**	**1.11 (1.06 to 1.16)**
Inpatient out-of-pocket costs	741 (2,328)	672 (2,083)	51 (-42 to 136)	1.08 (0.94 to 1.21)
Outpatient out-of-pocket costs	1,342 (1,662)	1,372 (2,010)	-33 (-91 to 31)	0.98 (0.93 to 1.05)
Prescription drug out-of-pocket drug costs	778 (850)	518 (678)	254 (223 to 280)	1.77 (1.66 to 1.89)
ARNI/ACE-I/ARBs	306 (399)	30 (111)	271 (260 to 281)	10.55 (9.53 to 11.85)
*Other prescription drugs**** *	472 (613)	488 (654)	-17 (-40 to 7)	0.97 (0.92 to 1.02)

*Censoring for death or disenrollment before 365 days was accounted for based on weighting by Kaplan-Meier probabilities as described by Lin et al.[23]
**Mean differences, mean ratios, and 95% CIs were calculated using a non-parametric bootstrapping method with 500 samples drawn with replacement.
***Total costs with and without rebates include home health services, which were not included in the inpatient, outpatient, or prescription drug costs.
**** All prescription drugs except sacubitril-valsartan, ACE-Is, or ARBs.

© 2025 Hwang CS et al. *JAMA Health Forum*

eTable 19. Total health care and out-of-pocket costs during the 365 days following sacubitril-valsartan or ACE-I/ARB initiation using an *intention-to-treat* approach for patients *with baseline health care costs in the SECOND LOWEST quartile*.

	Sacubitril-Valsartan Initiators, mean annual cost (SD)*	ACE-I/ARB Initiators, mean annual cost (SD)*	Mean Cost Difference (95% CI)**	Mean Cost Ratio (95% CI)**
**Total costs without sacubitril-valsartan rebates*** ($)**	**32,221 (35,874)**	**31,751 (39,322)**	**776 (-847 to 2,433)**	**1.03 (0.97 to 1.08)**
Inpatient costs	14,434 (29,407)	15,603 (33,018)	-970 (-2,397 to 429)	0.94 (0.85 to 1.03)
Outpatient costs	10,251 (13,542)	10,335 (13,544)	-8 (-525 to 498)	1.00 (0.95 to 1.05)
Prescription drug costs	5,758 (6,427)	3,855 (7,923)	1,876 (1,625 to 2,135)	1.50 (1.41 to 1.60)
ARNI/ACE-I/ARBs	857 (945)	166 (651)	1,811 (1,761 to 1,865)	12.63 (11.22 to 14.47)
*Other prescription drugs*** **	3,735 (5,557)	3,689 (7,864)	66 (-177 to 308)	1.02 (0.95 to 1.09)
**Total costs with sacubitril-valsartan rebates*** ($)**	**31,498 (35,835)**	**31,701 (39,311)**	**127 (-1,493 to 1,781)**	**1.00 (0.95 to 1.06)**
Inpatient costs	14,434 (29,407)	15,603 (33,018)	-970 (-2,397 to 429)	0.94 (0.85 to 1.03)
Outpatient costs	10,251 (13,542)	10,335 (13,544)	-8 (-525 to 498)	1.00 (0.95 to 1.05)
Prescription drug costs	5,036 (6,068)	3,805 (7,897)	1,228 (989 to 1,479)	1.33 (1.25 to 1.42)
Sacubitril-valsartan/ACE-I/ARBs	1,302 (1,177)	116 (415)	1,162 (1,130 to 1,197)	11.60 (10.40 to 13.13)
Other prescription drugs	3,735 (5,557)	3,689 (7,864)	66 (-177 to 308)	1.02 (0.95 to 1.09)
**Out-of-pocket costs ($)**	**3,628 (3,402)**	**3,454 (3,494)**	**174 (29 to 314)**	**1.05 (1.01 to 1.10)**
Inpatient out-of-pocket costs	986 (2,143)	992 (2,183)	1 (-109 to 96)	1.00 (0.90 to 1.10)
Outpatient out-of-pocket costs	1,785 (2,050)	1,815 (2,142)	-49 (-128 to 26)	0.97 (0.93 to 1.02)
Prescription drug out-of-pocket drug costs	857 (945)	646 (870)	222 (189 to 256)	1.36 (1.30 to 1.42)
ARNI/ACE-I/ARBs	282 (394)	32 (120)	244 (234 to 256)	9.25 (8.29 to 10.56)
*Other prescription drugs*** **	575 (697)	614 (845)	-22 (-53 to 6)	0.96 (0.92 to 1.01)

*Censoring for death or disenrollment before 365 days was accounted for based on weighting by Kaplan-Meier probabilities as described by Lin et al.[23]
**Mean differences, mean ratios, and 95% CIs were calculated using a non-parametric bootstrapping method with 500 samples drawn with replacement.
***Total costs with and without rebates include home health services, which were not included in the inpatient, outpatient, or prescription drug costs.
**** All prescription drugs except sacubitril-valsartan, ACE-Is, or ARBs.

© 2025 Hwang CS et al. *JAMA Health Forum*

eTable 20. Total health care and out-of-pocket costs during the 365 days following sacubitril-valsartan or ACE-I/ARB initiation using an *intention-to-treat* approach for patients *with baseline health care costs in the SECOND HIGHEST quartile*.

	Sacubitril-Valsartan Initiators, mean annual cost (SD)*	ACE-I/ARB Initiators, mean annual cost (SD)*	Mean Cost Difference (95% CI)**	Mean Cost Ratio (95% CI)**
**Total costs without sacubitril-valsartan rebates*** ($)**	**40,397 (44,469)**	**37,746 (44,841)**	**1,915 (70 to 3,762)**	**1.05 (1.00 to 1.10)**
Inpatient costs	19,578 (36,371)	19,028 (36,424)	-77 (-1,793 to 1,482)	1.00 (0.91 to 1.08)
Outpatient costs	11,657 (15,116)	11,846 (17,061)	-149 (-766 to 520)	0.99 (0.93 to 1.05)
Prescription drug costs	6,800 (9,581)	4,611 (7,979)	2,148 (1,789 to 2,472)	1.49 (1.38 to 1.58)
ARNI/ACE-I/ARBs	1,910 (1,849)	140 (601)	1,694 (1,644 to 1,748)	13.35 (11.45 to 15.59)
*Other prescription drugs**** *	4,889 (8,883)	4,472 (7,892)	451 (74 to 770)	1.11 (1.02 to 1.19)
**Total costs with sacubitril-valsartan rebates*** ($)**	**39,710 (44,443)**	**37,706 (44,831)**	**1,199 (-925 to 3,121)**	**1.03 (0.98 to 1.08)**
Inpatient costs	19,578 (36,371)	19,028 (36,424)	-77 (-1,793 to 1,482)	1.00 (0.91 to 1.08)
Outpatient costs	11,657 (15,116)	11,846 (17,061)	-149 (-766 to 520)	0.99 (0.93 to 1.05)
Prescription drug costs	6,113 (9,295)	4,572 (7,945)	1,537 (1,200 to 1,872)	1.35 (1.27 to 1.44)
Sacubitril-valsartan/ACE-I/ARBs	1,223 (1,182)	100 (389)	1,084 (1,050 to 1,123)	12.04 (10.50 to 13.85)
Other prescription drugs	4,889 (8,883)	4,472 (7,892)	451 (74 to 770)	1.11 (1.02 to 1.19)
**Out-of-pocket costs ($)**	**4,317 (4,397)**	**4,206 (5,497)**	**173 (-2 to 348)**	**1.04 (1.00 to 1.09)**
Inpatient out-of-pocket costs	1,346 (2,955)	1,302 (4,019)	86 (-55 to 227)	1.07 (0.96 to 1.19)
Outpatient out-of-pocket costs	2,136 (2,582)	2,274 (3,170)	-119 (-241 to 2)	0.95 (0.89 to 1.00)
Prescription drug out-of-pocket drug costs	835 (1,040)	630 (863)	215 (175 to 252)	1.35 (1.28 to 1.43)
ARNI/ACE-I/ARBs	243 (368)	23 (90)	215 (205 to 225)	10.41 (9.05 to 11.79)
*Other prescription drugs**** *	592 (830)	607 (845)	-4 (-35 to 29)	0.99 (0.94 to 1.05)

*Censoring for death or disenrollment before 365 days was accounted for based on weighting by Kaplan-Meier probabilities as described by Lin et al.[23]

**Mean differences, mean ratios, and 95% CIs were calculated using a non-parametric bootstrapping method with 500 samples drawn with replacement.

***Total costs with and without rebates include home health services, which were not included in the inpatient, outpatient, or prescription drug costs.

**** All prescription drugs except sacubitril-valsartan, ACE-Is, or ARBs.

© 2025 Hwang CS et al. *JAMA Health Forum*

eTable 21. Total health care and out-of-pocket costs during the 365 days following sacubitril-valsartan or ACE-I/ARB initiation using an *intention-to-treat* approach for patients *with baseline health care costs in the HIGHEST quartile*.

	Sacubitril-Valsartan Initiators, mean annual cost (SD)*	ACE-I/ARB Initiators, mean annual cost (SD)*	Mean Cost Difference (95% CI)**	Mean Cost Ratio (95% CI)**
**Total costs without sacubitril-valsartan rebates*** ($)**	**58,419 (62,017)**	**57,611 (59,150)**	**-204 (-3,086 to 2,705)**	**1.00 (0.95 to 1.05)**
Inpatient costs	28,085 (46,085)	30,166 (45,930)	-1,895 (-4,265 to 473)	0.94 (0.86 to 1.02)
Outpatient costs	17,433 (28,282)	17,159 (23,173)	-619 (-1,703 to 365)	0.96 (0.90 to 1.02)
Prescription drug costs	9,859 (21,986)	7,140 (16,861)	2,491 (1,628 to 3,251)	1.37 (1.22 to 1.52)
ARNI/ACE-I/ARBs	1,771 (1,829)	113 (511)	1,547 (1,492 to 1,606)	15.43 (12.88 to 18.34)
*Other prescription drugs***	7,026 (16,822)	7,026 (16,822)	948 (60 to 1,806)	1.15 (1.01 to 1.29)
**Total costs with sacubitril-valsartan rebates*** ($)**	**57,782 (61,950)**	**57,580 (59,145)**	**-701 (-3,610 to 2,154)**	**0.99 (0.94 to 1.04)**
Inpatient costs	28,085 (46,085)	30,166 (45,930)	-1,895 (-4,265 to 473)	0.94 (0.86 to 1.02)
Outpatient costs	17,433 (28,282)	17,159 (23,173)	-619 (-1,703 to 365)	0.96 (0.90 to 1.02)
Prescription drug costs	9,222 (21,795)	7,109 (16,847)	1,945 (1,062 to 2,712)	1.29 (1.14 to 1.44)
Sacubitril-valsartan/ACE-I/ARBs	1,134 (1,172)	82 (327)	994 (960 to 1,033)	13.76 (11.64 to 15.95)
Other prescription drugs	8,088 (21,507)	7,026 (16,822)	948 (60 to 1,806)	1.15 (1.01 to 1.29)
**Out-of-pocket costs ($)**	**6,245 (7,766)**	**6,198 (7,975)**	**-96 (-419 to 241)**	**0.98 (0.93 to 1.04)**
Inpatient out-of-pocket costs	2,025 (4,209)	2,165 (4,791)	-57 (-283 to 155)	0.97 (0.88 to 1.08)
Outpatient out-of-pocket costs	3,436 (5,822)	3,436 (5,822)	-234 (-483 to -10)	0.93 (0.86 to 1.00)
Prescription drug out-of-pocket drug costs	185 (325)	19 (89)	196 (144 to 244)	1.33 (1.23 to 1.43)
ARNI/ACE-I/ARBs	817 (1429)	597 (1,064)	162 (152 to 171)	9.65 (7.84 to 11.63)
*Other prescription drugs***	633 (1297)	577 (1,047)	35 (-14 to 84)	1.06 (0.98 to 1.15)

*Censoring for death or disenrollment before 365 days was accounted for based on weighting by Kaplan-Meier probabilities as described by Lin et al.[23]
**Mean differences, mean ratios, and 95% CIs were calculated using a non-parametric bootstrapping method with 500 samples drawn with replacement.
***Total costs with and without rebates include home health services, which were not included in the inpatient, outpatient, or prescription drug costs.
**** All prescription drugs except sacubitril-valsartan, ACE-Is, or ARBs.

© 2025 Hwang CS et al. *JAMA Health Forum*

eTable 22. Total health care and out-of-pocket costs during the 365 days following sacubitril-valsartan or ACE-I/ARB initiation using an *AS-TREATED* approach as sensitivity analyses.

	Sacubitril-Valsartan Initiators, mean annual cost (SD)*	ACE-I/ARB Initiators, mean annual cost (SD)*	Mean Cost Difference (95% CI)**	Mean Cost Ratio (95% CI)**
**Total costs without sacubitril-valsartan rebates*** ($)**	**21,452 (28,810)**	**22,375 (31,007)**	**-1,796 (-2,346 to -1,309)**	**0.90 (0.87 to 0.92)**
Inpatient costs	8,802 (19,891)	10,414 (22,630)	-1,699 (-2,060 to -1,351)	0.80 (0.76 to 0.84)
Outpatient costs	7,131 (13,277)	7,737 (13,758)	-682 (-903 to -470)	0.88 (0.85 to 0.92)
Prescription drug costs	4,389 (9,281)	2,895 (8,484)	797 (679 to 918)	1.38 (1.31 to 1.45)
ARNI/ACE-I/ARBs	*1,508 (1,805)*	*47 (147)*	*931 (910 to 950)*	*27.64 (26.09 to 29.20)*
*Other prescription drugs*** **	*2,881 (8,562)*	*2,848 (8,477)*	*-135 (-249 to -21)*	*0.94 (0.88 to 0.99)*
**Total costs with sacubitril-valsartan rebates*** ($)**	**20,915 (28,605)**	**22,367 (31,008)**	**-2,127 (-2,671 to -1,638)**	**0.88 (0.85 to 0.90)**
Inpatient costs	8,802 (19,891)	10,414 (22,630)	-1,699 (-2,060 to -1,309)	0.80 (0.76 to 0.84)
Outpatient costs	7,131 (13,277)	7,737 (13,758)	-682 (-903 to -470)	0.88 (0.85 to 0.92)
Prescription drug costs	3,852 (8,988)	2,887 (8,484)	466 (347 to 585)	1.22 (1.16 to 0.29)
Sacubitril-valsartan/ACE-I/ARBs	*970 (1,161)*	*39 (105)*	*600 (587 to 613)*	*21.89 (20.75 to 23.04)*
Other prescription drugs	*2,881 (8,562)*	*2,848 (8,477)*	*-135 (-249 to -21)*	*0.94 (0.88 to 0.99)*
**Out-of-pocket costs ($)**	**2,342 (3,258)**	**2,428 (3,827)**	**-156 (-212 to -107)**	**0.91 (0.89 to 0.94)**
Inpatient out-of-pocket costs	529 (1,318)	585 (1,732)	-62 (-89 to -39)	0.86 (0.81 to 0.91)
Outpatient out-of-pocket costs	1,272 (2,524)	1,444 (3,032)	-172 (-210 to -135)	0.84 (0.81 to 0.87)
Prescription drug out-of-pocket drug costs	541 (876)	399 (701)	78 (66 to 89)	1.27 (1.22 to 1.31)
ARNI/ACE-I/ARBs	*194 (348)*	*13 (35)*	*113 (109 to 116)*	*13.13 (12.45 to 13.85)*
*Other prescription drugs*** **	*347 (650)*	*386 (693)*	*-35 (-46 to -26)*	*0.87 (0.84 to 0.91)*

*Censoring for death or disenrollment before 365 days was accounted for based on weighting by Kaplan-Meier probabilities as described by Lin et al.[23]
**Mean differences, mean ratios, and 95% CIs were calculated using a non-parametric bootstrapping method with 500 samples drawn with replacement.
***Total costs with and without rebates include home health services, which were not included in the inpatient, outpatient, or prescription drug costs.
**** All prescription drugs except sacubitril-valsartan, ACE-Is, or ARBs.

eTable 23. Censoring reasons for our 13,775 matched pairs in our *AS-TREATED* sensitivity analyses.

Reason for censoring	N (%)
Death	2,202 (8.00)
Disenrollment	6,438 (23.40)
End of 365-day follow-up period	4,400 (15.99)
Switched to an ARNI or ACE-I/ARB in the opposite group*	1,253 (4.55)
Discontinued medication*	13,217 (48.04)

*30-day grace period between prescriptions allowed

© 2025 Hwang CS et al. *JAMA Health Forum*

eTable 24. Total and out-of-pocket spending during the 365 days following sacubitril-valsartan or ACE-I/ARB initiation. Sensitivity analyses using 1,000 BOOTSTRAPS, compared to using 500 bootstraps.

	Mean Cost Difference (95% CI)*		Mean Cost Ratio (95% CI)*	
	**500 bootstraps****	**1,000 bootstraps****	**500 bootstraps****	**1,000 bootstraps****
**Total costs <u>without</u> sacubitril-valsartan rebates*** ($)**	**701 (-132 to 1,593)**	**703 (-148 to 1,603)**	**1.02 (1.00 to 1.05)**	**1.02 (1.00 to 1.05)**
Inpatient costs	-790 (-1,468 to -72)	-793 (-1,442 to -82)	0.96 (0.92 to 1.00)	0.96 (0.92 to 1.00)
Outpatient costs	-330 (-664 to -11)	-328 (-675 to 14)	0.97 (0.94 to 1.00)	0.97 (0.94 to 1.00)
Prescription drug costs	1,911 (1,704 to 2,113)	1,915 (1,713 to 2,123)	1.45 (1.39 to 1.52)	1.45 (1.39 to 1.52)
ARNI/ACE-I/ARBs	*1,761 (1,736 to 1,788)*	*1,761 (1,736 to 1,787)*	*14.24 (13.34 to 15.35)*	*14.24 (13.34 to 15.29)*
*Other prescription drugs*** **	*150 (-61 to 344)*	*154 (-47 to 353)*	*1.04 (0.99 to 1.09)*	*1.04 (0.99 to 1.09)*
**Total costs <u>with</u> sacubitril-valsartan rebates*** ($)**	**70 (-761 to 956)**	**73 (-782 to 975)**	**1.00 (0.98 to 1.03)**	**1.00 (0.98 to 1.03)**
Inpatient costs	-790 (-1,468 to -72)	-793 (-1,442 to -82)	0.96 (0.92 to 1.00)	0.96 (0.92 to 1.00)
Outpatient costs	-330 (-664 to -11)	-328 (-675 to 14)	0.97 (0.94 to 1.00)	0.97 (0.94 to 1.00)
Prescription drug costs	1,281 (1,076 to 1,479)	1,285 (1,081 to 1,487)	1.31 (1.25 to 1.36)	1.31 (1.25 to 1.37)
ARNI/ACE-I/ARBs	*1,130 (1,114 to 1,147)*	*1,130 (1,114 to 1,147)*	*12.84 (12.08 to 13.75)*	*12.84 (12.08 to 13.69)*
*Other prescription drugs*** **	*150 (-61 to 344)*	*154 (-47 to 353)*	*1.04 (0.99 to 1.09)*	*1.04 (0.99 to 1.09)*
**Out-of-pocket costs ($)**	**109 (13 to 208)**	**110 (16 to 210)**	**1.03 (1.00 to 1.06)**	**1.03 (1.00 to 1.06)**
Inpatient out-of-pocket costs	9 (-51 to 64)	10 (-52 to 73)	1.01 (0.96 to 1.05)	1.01 (0.96 to 1.06)
Outpatient out-of-pocket costs	-129 (-199 to -67)	-129 (-195 to -66)	0.94 (0.91 to 0.97)	0.94 (0.91 to 0.97)
Prescription drug out-of-pocket drug costs	229 (211 to 246)	229 (211 to 246)	1.40 (1.36 to 1.43)	1.40 (1.36 to 1.44)
ARNI/ACE-I/ARBs	*228 (223 to 233)*	*228 (224 to 233)*	*10.10 (9.44 to 10.78)*	*10.09 (9.43 to 10.78)*
*Other prescription drugs*** **	*0 (-16 to 16)*	*1 (-16 to 16)*	*1.00 (0.97 to 1.03)*	*1.00 (0.97 to 1.03)*

*Positive mean cost differences indicate that sacubitril-valsartan initiators incurred higher spending, whereas negative mean cost differences indicate that ACE-I/ARB initiators incurred higher spending. Similarly, mean cost ratios greater than 1.0 indicate that sacubitril-valsartan initiators incurred higher spending, whereas mean cost ratios less than 1.0 indicate that ACE-I/ARB initiators incurred higher spending. Censoring for death or disenrollment before 365 days was accounted for based on weighting by Kaplan-Meier probabilities as described by Lin et al.[23]

** Mean differences, mean ratios, and 95% CIs comparing sacubitril-valsartan initiators to ACE-I/ARB initiators were calculated using a non-parametric bootstrapping method with 500 samples drawn with replacement.

***Total costs without rebates include home health services, which were not included in the inpatient, outpatient, or prescription drug costs.

**** All prescription drugs <u>except</u> sacubitril-valsartan, ACE-Is, or ARBs.

© 2025 Hwang CS et al. *JAMA Health Forum*

eFigure 1. Propensity scores by exposure group for all patients prior to matching.

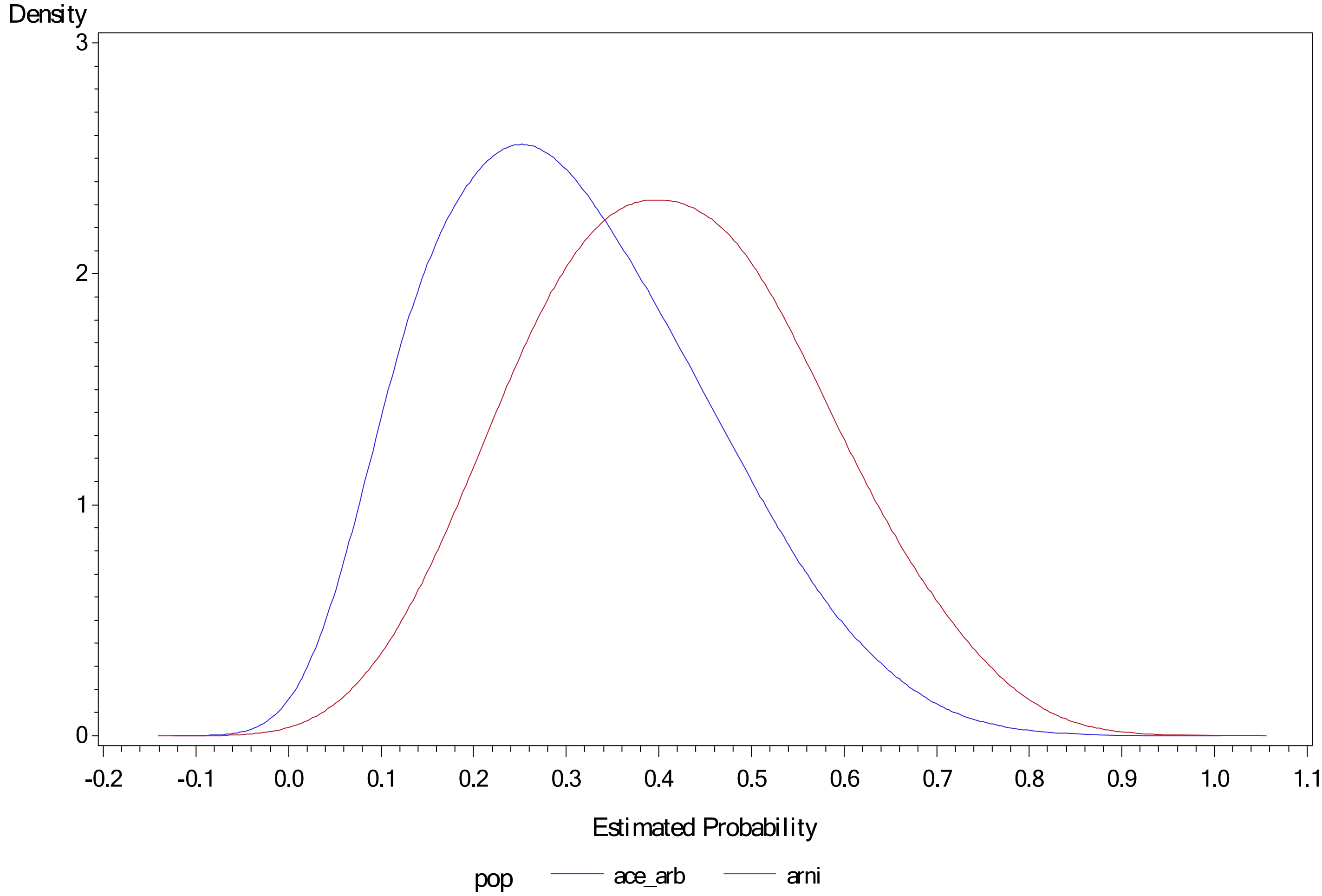

© 2025 Hwang CS et al. *JAMA Health Forum*

eFigure 2. Propensity scores by exposure group for the matched cohort.

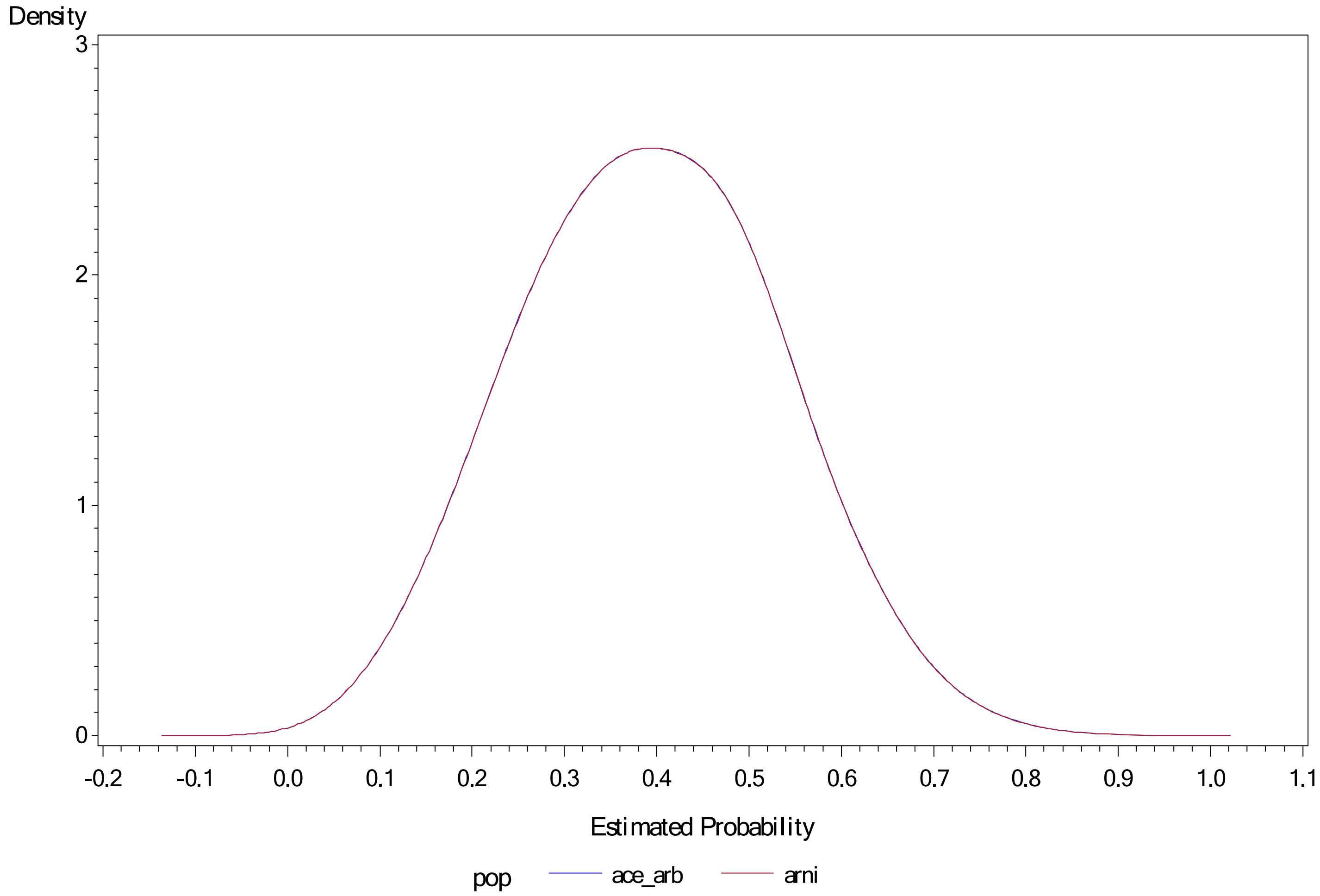

© 2025 Hwang CS et al. *JAMA Health Forum*
