# Supplementary material for: Health Care Spending After Initiating Sacubitril-Valsartan vs Renin-Angiotensin System Blockers for Heart Failure Treatment
Source: JAMA Health Forum. 2025 Feb 14;6(2):e245385. doi: 10.1001/jamahealthforum.2024.5385 (PMC11829231; doi:10.1001/jamahealthforum.2024.5385)
Supplement: Supplement 2. — Data Sharing Statement [file jamahealthforum-e245385-s002.pdf]

Data Sharing Statement

Hwang. Health Care Spending After Initiating Sacubitril-Valsartan vs Renin-Angiotensin System Blockers for Heart Failure Treatment. *JAMA Health Forum*. Published February 14, 2025. doi:10.1001/jamahealthforum.2024.5385

Data
Data available: No

Additional Information
Explanation for why data not available: We are unable to share patient-level data due to our data use agreement. However, the data can be purchased directly from the Center for Medicare and Medicaid Services. The analytic code is available upon request.
